# Non-Coding RNA Biomarkers in Male Infertility: From Discovery to Clinical Actionability—A Narrative Review

**DOI:** 10.3390/genes17091074

**Published:** 2026-09-06

**Authors:** Aris Kaltsas, Eleftheria Markou, Athanasios Zachariou, Fotios Dimitriadis, Nikolaos Sofikitis

**Affiliations:** 1 Third Department of Urology, Attikon University Hospital, School of Medicine, National and Kapodistrian University of Athens, 12462 Athens, Greece; akaltsas@med.uoa.gr; 2 Department of Microbiology, University Hospital of Ioannina, 45500 Ioannina, Greece; eleftheria.markou4@gmail.com; 3 Department of Urology, Faculty of Medicine, School of Health Sciences, University of Ioannina, 45110 Ioannina, Greece; zahariou@otenet.gr; 4 Department of Urology, Faculty of Medicine, School of Health Sciences, Aristotle University of Thessaloniki, 54124 Thessaloniki, Greece; helabio@yahoo.gr

**Keywords:** male infertility, non-coding RNA, microRNA, seminal plasma, extracellular vesicles, non-obstructive azoospermia, sperm retrieval, biomarker validation, clinical utility, external validation

## Abstract

Non-coding RNAs (ncRNAs) are biologically plausible biomarkers in male infertility, but no assay is ready for routine use. This narrative review organizes human evidence by intended clinical decision and defines clinical actionability as a test’s ability to inform a specified decision through analytical reliability, clinical validity, incremental value, decision-level benefit, and feasible implementation. Evidence is most developed for obstructive versus non-obstructive azoospermia (NOA) classification and sperm-retrieval prognosis. Most reports, however, use selected case–control samples, single-center development cohorts, or same-program evaluations. Mixed biospecimens, incompletely specified RNA isoforms and normalization, uncertain cohort independence, imperfect diagnostic references, and protocol-dependent retrieval outcomes limit transportability. No independent geographic validation of a locked ncRNA assay or prospective evaluation of ncRNA-guided management was identified within the retrieved sources. Current guidelines do not recommend routine ncRNA testing. Progress requires prespecified intended uses, locked assays, representative multicenter validation, same-patient comparison with contemporary care, calibrated risk estimates, decision-curve analysis, and patient-important, couple-centered outcomes.

## 1. Introduction

Male-associated factors contribute to about half of infertility among couples [1,2]. Azoospermia should be confirmed on at least two centrifuged semen samples and distinguished from cryptozoospermia [3]. Obstructive azoospermia (OA) and non-obstructive azoospermia (NOA) require different management, but they are clinically useful categories rather than perfectly separable biological states. Partial obstruction, mixed pathology, and focal spermatogenesis create equivocal cases in which reference assignment matters. Infertility is a couple-level condition; male biomarkers must be interpreted with partner and treatment factors.

Guideline-based evaluation includes history, examination, semen analysis, hormonal assessment, and phenotype-directed genetic testing [2,3,4]. Exome sequencing is entering selected severe or idiopathic cases, but yield depends on phenotype, ancestry, gene set, pipeline, and variant-classification criteria. In a selected Estonian cohort, likely causal findings were reported in 64 of 521 men with primary spermatogenic failure and 20 of 185 men with NOA [5,6]. These estimates do not represent unselected populations. Neither current European Association of Urology (EAU) nor American Urological Association/American Society for Reproductive Medicine (AUA/ASRM) guidance recommends routine non-coding RNA (ncRNA) testing [2,3].

No single preoperative factor reliably predicts surgical sperm retrieval in NOA [3]. Complete deletions of azoospermia factor (AZF) regions a and b predict negligible retrieval, whereas complete AZFc deletions remain compatible with retrieval [3,4]. A meta-analysis of 3807 men found no significant association between AZFc deletion and retrieval [7]. Across 48 studies of Klinefelter syndrome, median retrieval was 44%, and mean rates did not differ significantly between adolescents and adults (45 vs. 42%) [8]. These data do not establish a benefit from adolescent retrieval or universal age independence. No validated ncRNA assay directs management in otherwise unexplained infertility [3].

Conventional semen categories are descriptive phenotypes rather than diagnoses of fertility or infertility. Some men have concentration, motility, and morphology within reference ranges despite failure to conceive, while abnormal parameters do not determine a couple’s outcome in isolation. Genetic prognosis is also endpoint-specific: a result that informs retrieval may not predict fertilization, embryo development, pregnancy, or live birth. A biomarker must therefore name both the intended population and the exact decision-linked endpoint.

ncRNAs are plausible analytes because spermatogenesis depends heavily on post-transcriptional regulation. MicroRNAs (miRNAs) regulate transcript stability and translation. PIWI-interacting RNAs (piRNAs) support transposon repression and germline integrity [9,10], while long non-coding RNAs (lncRNAs) and circular RNAs (circRNAs) have diverse regulatory roles [11,12]. Animal experiments support small-RNA remodeling during epididymal transit and effects on early development [13,14,15,16]. Human evidence, including human in vitro fertilization (IVF) studies, remains predominantly associative [17,18].

Accessible specimens include spermatozoa, whole semen, seminal plasma, seminal-plasma extracellular-vesicle (EV)-enriched fractions, and blood, but these compartments are not interchangeable. Seminal plasma has mixed reproductive-tract origins and is, therefore, a candidate proxy rather than an established liquid biopsy of testicular function [19]. Extracellular miRNAs may be vesicle-encapsulated or protein-bound, and carrier distribution depends on the analyte and fluid [20,21]. In this review, clinical actionability means that a defined result can inform a specified decision. Actionability requires analytical reliability, clinical validity, incremental value relative to current care, benefit at relevant thresholds, and feasible implementation.

This definition separates measurability from use. A stable association may support clinical validity without showing that the result improves counseling or management. An add-on marker must contribute information beyond the same clinical variables already available to the treating team. A replacement or triage test may instead justify use through lower invasiveness, faster delivery, or better access, provided that its residual error is acceptable for the intended decision.

Previous reviews have cataloged candidates by RNA class or biological mechanism [11,12,22,23,24]. That organization can obscure whether evidence supports a specific clinical decision. Persistent uncertainty despite increasing discovery suggests that study design, measurement architecture, and comparator choice, rather than candidate volume alone, limit translation.

A decision-centered narrative design was chosen to compare intended uses while integrating two cross-cutting problems: specification of the measurand and dependence of labels and retrieval outcomes on reference and procedural protocols.

The framework also prevents biological plausibility from being mistaken for clinical evidence. A molecule may have a compelling role in spermatogenesis yet remain unsuitable as a biomarker because the accessible compartment is indirect, the assay is unstable, or the proposed result does not alter a real decision. Conversely, a clinically informative signal need not establish the causal mechanism responsible for that signal.

The aim was to characterize human evidence by intended use and define the requirements separating promising discrimination from clinical actionability.

## 2. Materials and Methods

### 2.1. Review Design and Scope

This is a narrative review with purposive rather than systematic selection. The design was chosen for conceptual synthesis across clinical decisions and cross-cutting measurement and reference-standard problems, not exhaustive enumeration, pooled effect estimation, or formal study-level risk-of-bias assessment. Six intended uses were considered: infertility subtyping, OA/NOA classification, retrieval prognosis, assessment beyond conventional semen analysis, assisted-reproduction outcomes, and exploratory monitoring or other emerging applications.

The scope was restricted to clinical biomarker translation. Direct evidence comprised human studies linking an ncRNA analyte or panel to a male-infertility decision or outcome. Mechanistic, animal and in vitro studies were used only as biological context. Routine-variable models, protein or DNA markers, and mixed or predominantly coding sperm-RNA signatures were treated as contextual comparators and did not determine ncRNA evidence maturity.

The Scale for the Assessment of Narrative Review Articles (SANRA) informed drafting but was not used as a conduct or reporting guideline, and no score was assigned [25]. Selection was purposive, screening was not duplicated, and no quantitative synthesis was undertaken; the work is, therefore, reported as a narrative review without a claim of systematic or scoping-review methodology.

### 2.2. Bibliographic Search

PubMed/MEDLINE was searched from inception through 13 August 2026 with three concept blocks covering male-infertility phenotypes or retrieval procedures, ncRNA classes, and biomarker or reproductive outcomes. The exact specificity-oriented query is reproduced verbatim in Appendix A and returned 883 records on the execution date. Because selection was purposive, it is not a systematic-review screening denominator. Titles and abstracts were screened for clinically informative human studies, supplemented by targeted searches and citation tracking.

The submitted term for tRNA-derived fragments ((tRFs), tRF*[tiab]), did not truncate because PubMed requires four characters before a wildcard. On 31 August 2026, the original date-capped query returned 886 dynamically indexed records; replacing that term with tRF[tiab] OR tRFs[tiab] returned 887. The single added record was an irrelevant mouse Trf2 study. A subset of the corrected structured query covering tRFs and tRNA-derived small RNAs (tsRNAs) returned 35 records. A broader targeted follow-up returned 51, including 17 not retrieved by the submitted query; title screening found no additional decision-relevant human report. All four human tRF studies discussed in the review were retrieved by the submitted query. Appendix A provides the exact sensitivity strings and results.

Archived exports dated 24 July 2026 contained 1967 PubMed, 3038 Embase, and 2587 Scopus records. Because the exact Embase and Scopus query versions that generated these files could not be linked unambiguously, the exports were used only for a retrospective coverage audit, not as formal reproducible searches. DOI matching located 22, 21, and 19 of the 23 reports in the submitted Supplementary Table S1; the audit did not screen every exported record. Absence statements were consequently reduced in strength and remain bounded by the retrieved sources.

The archived files demonstrate that broader discovery work had occurred, but they cannot be converted retrospectively into the executed search for this review. Their deduplication history belongs to an earlier scoping workflow, and deterministic linkage did not reproduce the historical unique-record count exactly. The present revision therefore reports file provenance and coverage only. This approach preserves useful evidence about database breadth without implying a new systematic search or a PRISMA-compatible screening process.

### 2.3. Trial-Registry Searches

ClinicalTrials.gov, ISRCTN, and the World Health Organization (WHO) International Clinical Trials Registry Platform (ICTRP) were searched on 13 August 2026 with the exact strings in Appendix B. Six ClinicalTrials.gov, no ISRCTN, and three WHO ICTRP records were returned. Registrations without posted results were treated as ongoing evidence, not completed validation, and registry searches were not substitutes for bibliographic databases.

### 2.4. Study Selection and Data Collection

A decision-relevant study linked a human ncRNA analyte or panel or a prespecified contextual comparator to one intended use and reported enough information to characterize the population, specimen, assay, reference or outcome, and translational role. Diagnostic or prediction metrics were prioritized but not required. Association-only studies were retained as discovery evidence and were not treated as validated tests. Studies were not excluded solely because the area under the receiver operating characteristic curve (AUC), sensitivity, or specificity was unavailable. Exclusively mechanistic or animal studies were confined to brief context.

Screening and selection were performed by A.K.; E.M. checked structured descriptors and quantitative claims against primary reports. For each decision-relevant study, design, setting, population, specimen, assay, normalization, reference or outcome, threshold handling, performance, comparator, validation stage, and cohort provenance were recorded. Discordant findings were retained and interpreted according to design, compartment, outcome, and independence rather than vote counting. Reports with unresolved participant overlap were grouped as potentially overlapping and not counted as independent replication.

Development denoted performance estimated after feature, model, or threshold selection in the same data. Internal validation included split sampling or resampling within one center or program. A separate sample was not labeled external when recruitment, preprocessing, or model selection remained within the originating program. Geographic or temporal external validation required a locked assay and analysis applied without redevelopment in a distinct intended-use population. Performance estimates and confidence intervals were reproduced only when reported by the primary study.

These operational rules make the selected evidence auditable but do not make retrieval exhaustive. Studies with limited reporting could still inform context, while absence from the structured table does not mean formal exclusion after duplicate screening. The table supports design comparison rather than an included-study count.

### 2.5. Framework for Appraisal and Synthesis

A statistically significant difference is not sufficient for actionability. Evidence was, therefore, appraised across five linked domains defined with Food and Drug Administration–National Institutes of Health Biomarkers, EndpointS, and other Tools (FDA–NIH BEST) terminology [26]. These are analytical validity, clinical validity, incremental value relative to prespecified current care, clinical utility at relevant thresholds, and implementation feasibility. Replacement or triage tests may offer value through lower burden, cost, or delay despite similar discrimination. These domains organize appraisal but do not impose one evidentiary threshold across different intended uses [27,28].

The Quality Assessment of Diagnostic Accuracy Studies, version 3 (QUADAS-3) informed diagnostic-accuracy descriptors, and the Prediction model Risk Of Bias ASsessment Tool + Artificial Intelligence (PROBAST + AI) informed prediction-model descriptors [29,30]. Neither instrument was applied item by item, and no numerical quality score was assigned. Reporting under the Minimum Information for Publication of Quantitative Real-Time PCR Experiments (MIQE) and the Minimal Information for Studies of Extracellular Vesicles (MISEV2023) was also not formally scored [31,32]. Supplementary Table S1 presents non-scored design and provenance descriptors appropriate to each report.

Findings were synthesized narratively by intended use. Pooling and cross-study ranking were avoided because measurands, recruitment spectra, prevalence, reference standards, outcome definitions, and validation designs were not sufficiently commensurate. Appraisal emphasized whether evidence arose from selected case–control contrasts or representative clinical cohorts and whether OA/NOA labels and retrieval outcomes were protocol-dependent.

Evidence-maturity labels were descriptive. Discovery denotes association or apparent performance without separate evaluation. Internal validation denotes split-sample, resampling, or same-center evaluation. Same-program validation denotes follow-up without established program independence. External validation requires a locked assay or model in an independent temporal or geographic population. Clinical utility requires evidence that test-guided care improves decisions, net benefit, or patient-important outcomes. These author appraisals are not scores. Statements that evidence was not identified refer only to the reported searches and are less secure than conclusions from exhaustive systematic retrieval.

BioRender AI (BioRender, Toronto, ON, Canada; web-based application with no displayed version identifier; accessed 16 August 2026) was used solely to draft the conceptual schematics in this review from author-written specifications. It was not used to generate or alter research data. All labels and scientific content were entered, reviewed, and verified by the authors against the cited sources. Further disclosure is provided in the Acknowledgments.

## 3. Clinical Pathway and Intended Uses

### 3.1. Loci of Diagnostic Uncertainty

Four decision points remain incompletely resolved after guideline-based evaluation. They concern equivocal OA/NOA classification, retrieval counseling in confirmed NOA, whether a male measure should alter assisted reproduction, and otherwise unexplained infertility despite unremarkable semen parameters. A new assay must be evaluated in the population facing the decision, not in clinically obvious comparison groups.

The retrieved literature did not provide a robust multicenter estimate of the proportion remaining equivocal after standard OA/NOA assessment. Clinical value, therefore, cannot be inferred from accuracy in clearly separated groups. OA/NOA classification has the most developed ncRNA evidence but may address a restricted residual population; retrieval prognosis addresses a major decision but lacks independent geographic ncRNA validation.

This denominator is clinically important. A classifier tested in post-vasectomy OA and severe secretory failure may separate biological extremes. It may add little value after routine history, examination, semen volume and pH, endocrine testing, genetics, and imaging have resolved most cases. Consecutive recruitment of genuinely equivocal patients is, therefore, more informative than another extreme-group comparison.

For each intended use, the result should also be located within the couple’s pathway. Better male-factor classification may improve counseling without changing treatment, whereas retrieval prognosis can influence whether surgery is attempted. Assisted reproductive technology (ART) biomarkers face another question: whether the result changes a choice not already determined by female, embryo, or laboratory factors.

### 3.2. Intended Uses Defined in Advance

Diagnostic, prognostic, predictive, and monitoring uses were distinguished [26]. Diagnosis classifies a present condition; prognosis estimates an outcome under specified management. A predictive biomarker identifies differential intervention benefit or harm and, therefore, requires comparative treatment data. Monitoring requires serial measurement and evidence that change is analytically and clinically interpretable; the retrieved monitoring evidence was hypothesis-generating.

For an add-on assay, incremental value requires a same-patient comparison of prespecified current care with and without ncRNA. A replacement or triage assay may instead combine non-inferior performance with lower burden, cost, or delay.

## 4. Biological and Analytical Foundations

### 4.1. ncRNA Classes Relevant to Male Reproduction

miRNAs direct post-transcriptional repression and are the most studied class [22]. piRNAs act with PIWI proteins in transposon repression [9,10]. Mouse microinjection experiments support the sufficiency of selected sperm tsRNA-enriched fractions for offspring metabolic phenotypes. They do not establish physiological necessity or human causality [33,34]. Long non-coding RNAs and circRNAs have diverse regulatory functions [11,12]. Functions of ribosomal RNA (rRNA)- and Y-RNA-derived fragments remain less resolved [20].

### 4.2. Roles Across the Reproductive Axis

These RNA classes participate across spermatogenesis. Animal studies support epididymal remodeling and effects of selected sperm RNAs on early development [13,14,15,16,33], whereas human evidence is mainly associative [17]. Standard poly(A)-capture single-cell atlases refine mRNA-defined testicular states but do not directly quantify miRNAs, piRNAs, or most tsRNAs [35,36,37]. Exposure-related variation is biologically plausible and may confound human studies [38,39,40].

### 4.3. Non-Interchangeable Compartments

Spermatozoa, semen cell pellets, seminal plasma, EV-enriched fractions, testicular tissue, blood, and urine are distinct compartments. Spermatozoa are unavailable in azoospermia, seminal plasma has mixed origins, and cell pellets require objective assessment of leukocyte and somatic-cell contamination [41,42,43]. A marker discovered in tissue is not thereby validated in seminal plasma.

Seminal plasma is dominated by accessory-gland secretions, with smaller epididymal and testicular contributions [19]. Its composition may therefore change with glandular function, inflammation, abstinence, and fractionation, even when testicular spermatogenesis is unchanged. EV-enriched fractions add another layer because isolation methods co-enrich different vesicular and non-vesicular particles. Cross-compartment transportability must be demonstrated empirically for each candidate.

### 4.4. Detection Platforms

Small-RNA sequencing, microarrays, reverse transcription quantitative polymerase chain reaction (RT-qPCR), droplet digital PCR, and biosensor prototypes differ in bias, specificity, and routine readiness. Library chemistry can markedly alter sequence representation [44]. In 25 sperm samples, panoramic RNA display by overcoming RNA modification aborted sequencing (PANDORA-seq) recovered modified tsRNA and rRNA-derived species under-represented by conventional sequencing [45]. A 70:30 split, however, reproduced semen-category labels rather than a clinical outcome. RT-qPCR may also quantify an isoform aggregate different from the sequencing feature [46]. Table 1 separates biological plausibility from human clinical evidence.

Discovery and verification platforms are not interchangeable by default. Sequencing may resolve a precise isoform or cleavage product, whereas a later amplification assay can capture several related molecules. A successful technical transfer therefore requires sequence-level identity, verified assay specificity, common controls, and evidence that the normalization strategy is stable across the intended phenotypes. Without these elements, apparent replication may concern a different quantity.

## 5. Clinical Evidence by Decision Point

### 5.1. Detection and Diagnostic Subtyping

Most human evidence uses selected case–control contrasts. Five sperm/testis miRNAs in 226 clinic attendees produced individual-marker AUCs of 0.777–0.988, but combined-panel performance was not reported [48]. Seminal-plasma piRNAs differed between 211 infertile men and 91 fertile controls [47], and tissue miRNAs differed across selected histological patterns [68]. These findings support discovery and subtyping hypotheses, not a decision-ready test.

Control definitions materially affect these contrasts. Men with semen parameters within reference ranges may still belong to infertile couples, whereas proven-fertility controls reflect a different target. Testicular tissue studies also condition participation on biopsy or surgery and cannot represent men evaluated non-invasively. The resulting effect sizes describe the sampled groups, not the probability that a future clinic patient has a management-relevant condition.

A pilot plasma miR-20a-5p study illustrates the accessibility of blood-based testing [69], but the peripheral compartment is distant from the testis and vulnerable to hemolysis. Normozoospermic clinic controls are also not equivalent to men of proven fertility; extreme-group sampling can overstate deployment performance.

### 5.2. Differential Diagnosis of Azoospermia

OA/NOA classification has the most developed diagnostic evidence. A seminal-plasma EV study associated miRNAs with azoospermia origin and testicular sperm presence [49]. A same-program follow-up evaluated miR-31-5p across two EV-enriched preparations and whole seminal plasma, reporting overall AUCs of 0.721–0.883 and higher values in a small subgroup with follicle-stimulating hormone (FSH) below 10 IU/L [50]. The OA group was dominated by post-vasectomy cases, and the comparator included secretory azoospermia or cryptozoospermia. These spectra do not establish performance in consecutive equivocal cases. Subsequent EV sequencing [51] and a plasma EV miRNA–FSH model [52] remain without independent geographic validation.

The miR-31-5p follow-up is informative because it tested a pre-analytical choice rather than assuming that ultracentrifugation, precipitation, and whole seminal plasma were equivalent. AUCs varied by preparation, and confidence intervals were not reported for the small FSH-defined subgroup [50]. This program advances assay architecture, but its same-program design and selected clinical spectrum leave transportability unresolved.

Contextual protein and spectroscopy studies have reported strong OA/NOA discrimination [70,71]. Their absence from current guidance illustrates that high initial accuracy does not establish clinical utility or routine implementation. Apparent perfect classification warrants particular scrutiny of recruitment spectrum, masking, and independent validation.

For example, a decision tree based on seminal extracellular matrix protein 1 (ECM1) and testis-expressed protein 101 (TEX101) reported 100% sensitivity and specificity for OA versus normal spermatogenesis at one threshold. Specificity for OA versus NOA was 73% [70]. A later seminal-plasma EV spectroscopy platform reported perfect OA/NOA accuracy [71]. These results define a demanding contextual benchmark while also showing why consecutive equivocal cases and blinded external evaluation are indispensable.

### 5.3. Sperm-Retrieval Prognosis in NOA

Retrieval prognosis addresses a concrete decision: counseling couples about the probability and consequences of proceeding with microdissection testicular sperm extraction (micro-TESE).

A nine-lncRNA seminal-plasma EV panel was developed after an 11-person discovery phase in 30 men with NOA and tested in a same-center holdout of 66 [59]. Reported AUCs were 0.99 and 0.96, with 93.5% sensitivity and 90.0% specificity in the holdout. Same-patient analyses favored the RNA panel over reduced clinical or hormone models but did not compare comprehensive current care with and without ncRNA. The small development set, same-center split, case-mix imbalance, β-actin-normalized preamplification, and absent calibration limit transportability.

The pooled 96-participant analyses reported an AUC 0.97 for the panel and 0.72 for a six-variable clinical model. In the 44 participants with inhibin-B data, corresponding AUCs were 0.96 and 0.69 for a three-hormone model [59]. These same-patient comparisons are useful, yet they do not isolate the added value of ncRNA because the complete clinical model was not refitted with and without the panel. Model calibration and threshold-specific net benefit were also unavailable.

Other retrieval studies remain predominantly single-center. Internal AUCs were 0.82–0.83 for piR-61927 in 45 men [54] and 0.96 for three circRNAs in a 52-person development cohort [61]. A four-miRNA internal test set produced an AUC of 0.93 [53]. Small plasma EV tRF studies reported AUCs of 0.89–0.95 [55,56]. A six-circRNA serum model reused a 20-sample screening subset within its 180-person evaluation dataset and reported an apparent AUC of 0.98 [62]. A tissue circ_MGLL nomogram used predictors available during or after micro-TESE and, therefore, cannot support preoperative triage [63].

Validation labels require attention in this literature. The three-circRNA model selected features after a six-tissue discovery stage but developed and evaluated the combined model in the same 52 men [61]. The four-miRNA study used a blinded internal test set after screening and training within one center [53]. The serum circRNA model drew its screening subset from the same 180-person dataset used for final evaluation [62]. These designs provide different protection against optimism and should not share one validation label.

Reported confidence intervals further illustrate uncertainty. The piR-61927 estimates had 95% confidence intervals of 0.63–1.00 and 0.66–1.00 in samples of 20 and 25 men [54]. The four-miRNA internal-test AUC of 0.93 lacked reported precision and calibration [53]. The two plasma tRF estimates came from only 30 men with NOA plus 12 fertile controls [56]. High point estimates from these samples are not precise clinical probabilities.

Intended uses must remain distinct: the tRF-Val-AAC-010 AUC of 0.96 concerned azoospermia origin, whereas retrieval AUC was 0.89 [55]. Four 2026 plasma RNA-axis reports from one author group described retrieval AUCs of 0.909–0.983 [64,65,66,72]. The first three reported the same 60-NOA/40-control cohort, whereas the accessible fourth report did not provide enough cohort detail to establish independence. These reports are grouped as one development program rather than independent replications. Other small-RNA and lncRNA reports remain hypothesis-generating [60,73,74].

The first three reports share ten authors and identical cohort totals, histological subgroups, retrieval counts, hormone summaries, blood volume, bilateral micro-TESE protocol, and ethics identifier [64,65,66]. They therefore represent one participant cohort analyzed through three molecular axes. The fourth report shares nine core authors, but its accessible abstract omits cohort fingerprints [72]. Its participant independence remains unresolved, so none of the four reports is counted as an independent replication of another.

**Figure 1:**
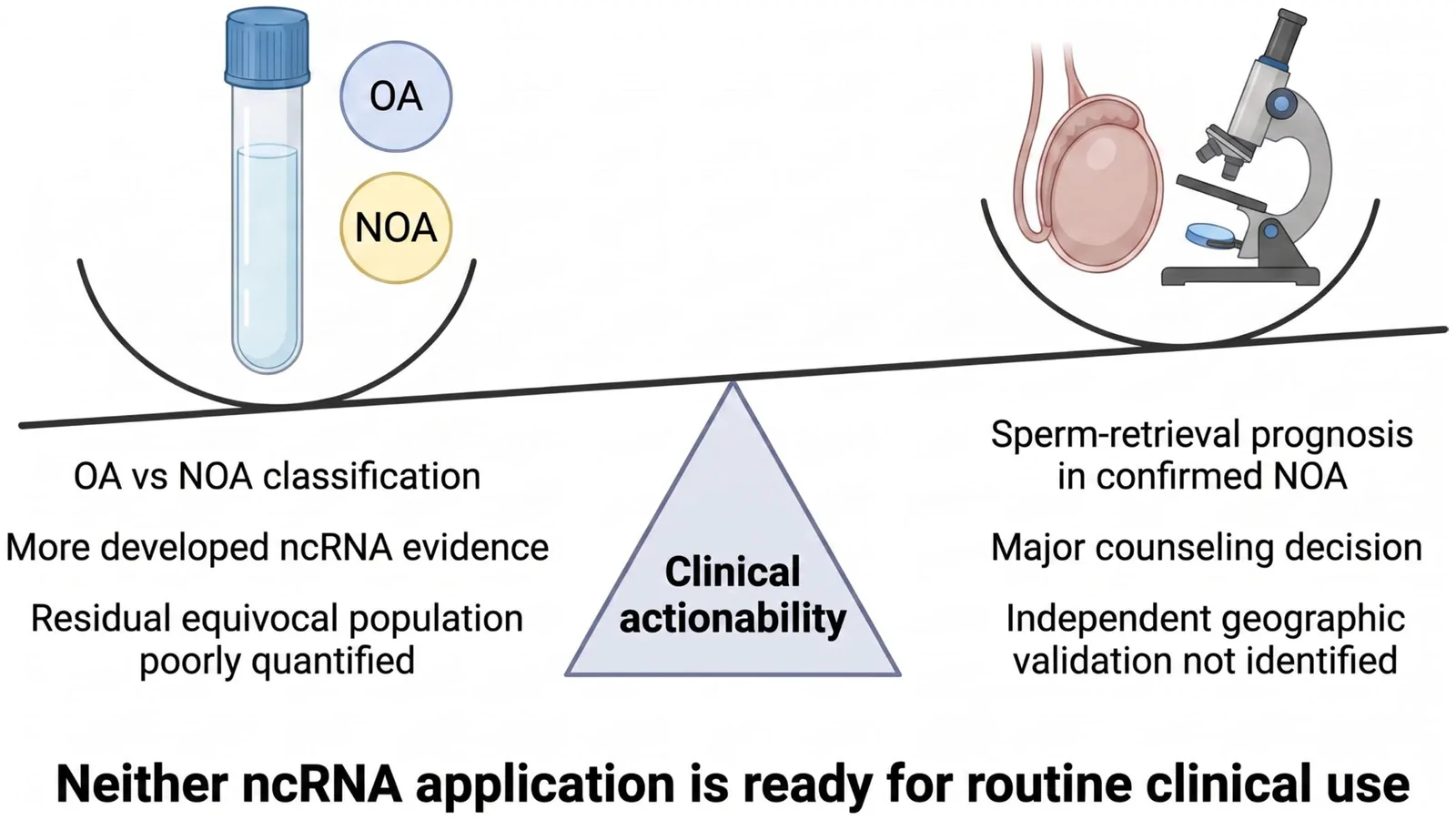
Evidence–need asymmetry of ncRNA biomarkers in azoospermia. OA/NOA classification has the more developed ncRNA evidence, but the residual population remaining equivocal after standard evaluation is poorly quantified. Retrieval prognosis addresses a major counseling decision in confirmed NOA, but independent geographic validation and biomarker-guided utility were not identified within the retrieved sources. Neither application is ready for routine care. This is a conceptual narrative representation, not pooled quantitative evidence or a formal evidence score. ncRNA, non-coding RNA; NOA, non-obstructive azoospermia; OA, obstructive azoospermia. Created in BioRender. Kaltsas, A. (2026) https://BioRender.com/wiqyymy, (accessed 16 August 2026).

Registry searches identified one completed observational program without posted results and one ongoing prospective circRNA study [75,76]. Neither provided completed evidence of ncRNA-guided care. Figure 1 summarizes the resulting evidence–need asymmetry.

### 5.4. Function Beyond Conventional Semen Analysis

Evidence beyond conventional semen analysis is sparse. A related 648-element sperm RNA signature was defined mainly by exonic elements in 96 couples, with 72 sequencing datasets passing quality control [77]. Observed live-birth proportions differed by signature completeness after timed intercourse or intrauterine insemination (IUI), but the retrospective association was not a causal treatment effect or validated rule. Because only 42 elements mapped to non-coding transcripts, this program is retained solely as contextual mixed-RNA evidence [77,78,79].

The signature was anchored in seven couples who achieved live birth after first-cycle timed intercourse. Among evaluable couples treated with timed intercourse or IUI, live-birth proportions were 73% with a complete element set and 27% when one or more elements were absent [77]. Treatment was not randomized, and approximately one-quarter of enrolled sequencing datasets failed quality control. These features limit causal and implementation inferences.

This setting lacks a single molecular reference standard. Reproductive outcomes are the most relevant anchors when couple-level determinants and treatment are modeled. Association with another laboratory measure, such as sperm DNA fragmentation, does not, by itself, establish clinical utility [80].

### 5.5. Prediction of Assisted-Reproduction Outcomes

Endpoints in this domain must be kept distinct: fertilization; cleavage and embryo quality; blastocyst formation; implantation, biochemical, clinical or ongoing pregnancy; and live birth, including cumulative live birth. These endpoints are not interchangeable; live birth—ideally cumulative healthy singleton live birth—is the most directly patient-important effectiveness endpoint, whereas fertilization, embryo development and biochemical pregnancy remain informative intermediate outcomes.

Evidence for ART outcomes remains exploratory and mainly concerns intermediate endpoints. Small-RNA sequencing in 54 donor-oocyte cycles compared selected high-, average-, and low-blastocyst-rate groups without locking a panel [81]. Two seminal tRF studies reported discordant ART associations and no externally validated rule [57,58]. Other reports associated sperm rRNA fragments or miRNAs with embryo development, pregnancy, or live birth but used small or retrospective cohorts and did not establish incremental value [67,82,83]. In the selected-sperm study, 39 libraries came from 13 donors and were not independent participant observations [83].

Fertilization, cleavage, embryo quality, blastocyst formation, implantation, biochemical pregnancy, clinical pregnancy, and live birth are not interchangeable endpoints. Donor oocytes reduce female-factor heterogeneity but also define a selected treatment population. Repeated embryos and cycles create clustering, while female age, ovarian response, laboratory practice, and embryo selection remain important covariates. A male marker should ultimately improve a couple-level decision, not merely correlate with an intermediate laboratory measure.

In the 54-man donor-oocyte study, analyses compared high, average, and low blastocyst-rate groups defined relative to the sample mean, and the report did not account fully for men outside those analyzed groups [81]. Another study prepared 39 selected-sperm libraries from only 13 discovery donors before evaluating bulk sperm in 85 men [83]. These units and measurand changes must be represented correctly in validation claims.

The tRF studies also show why titles and statistical results must be separated. A donor-oocyte intracytoplasmic sperm injection (ICSI) report associated three seminal tRFs with repeated failed cycles [57]. A later 48-man study found higher 5′tRF-Glu-CTC in oligozoospermia but no significant association with fertilization, embryo formation, or pregnancy [58]. Neither produced a locked rule, and the findings need not conflict because populations, endpoints, and assays differed.

Future ART studies should analyze couples and cycles, adjust for female and laboratory factors, and prioritize standardized patient-important outcomes such as cumulative live birth [22,84].

### 5.6. Emerging Applications

Varicocele, recurrent pregnancy loss, exposure, and monitoring findings remain hypothesis-generating [40,85,86,87,88,89,90,91]. Monitoring is especially difficult because abstinence, within-person biological variation, changing cellular or glandular contributions, assay drift, and treatment timing can mimic change. No retrieved study established a minimal-change threshold or showed that serial ncRNA results improved management. Table 2 distinguishes direct ncRNA evidence from contextual comparators.

Table 2 is intentionally selective. It emphasizes reports that clarify design, validation, cohort independence, or a decision-relevant outcome. Mechanistic and association studies remain cited in the narrative but do not contribute equally to maturity judgments. Contextual protein, routine-variable, and mixed-RNA reports are labeled because they define comparators or methodological lessons; they are not counted as evidence that an ncRNA assay is validated.

## 6. From Association to a Decision-Ready Test

High discrimination has not progressed to demonstrated ncRNA-guided utility or routine implementation. The remaining barriers concern measurement, design, comparator choice, and decision consequences.

### 6.1. Pre-Analytical Variability

Semen is analytically demanding. Abstinence affects semen parameters [98], processing determines captured cells and vesicles, contamination can dominate RNA preparations [41]; storage and freeze–thaw cycles alter recovery, and hemolysis confounds circulating miRNAs [99,100]. EV separation methods can also yield different RNA profiles [101].

A transportable protocol should define the abstinence interval, collection completeness, liquefaction time, processing delay, temperature, centrifugation, fractionation, storage, freeze–thaw limits, and contamination checks. Blood-based studies should specify serum or plasma, anticoagulant, platelet handling, and hemolysis assessment. Cases and controls should be balanced across extraction, library, and assay batches. Assay failures and repeat rules are part of performance rather than laboratory footnotes.

Future studies should balance extraction and assay batches, report failure rates, and assess repeatability across ejaculates. In 164 healthy men providing two samples, only 46 of 409 seminal EV and 9 of 265 sperm small ncRNAs met strict within-person variation and reliability criteria in a 10-pair sequencing subset [102]. This limited study cannot be generalized to infertile populations, but it shows that repeatability must be established for each analyte and compartment. EV-enriched preparations may also contain non-vesicular material [31,101].

Repeatability is particularly important for monitoring because a single person becomes the comparator over time. Biological variation must be separated from analytical imprecision before any change threshold is proposed. Studies also need to determine whether a repeat sample changes classification and whether that reclassification improves a decision. Without these data, serial measurement can add noise while appearing more informative than a single result.

### 6.2. The Underspecified Measurand

A molecular name alone does not define a measurand. A transportable result requires specification of the compartment, isolated fraction, RNA sequence and isoform boundaries, quantification chemistry, and normalization denominator.

Each component can change the result. The same molecular label may denote a canonical miRNA, one or more isomiRs, a precursor, or a family of tRNA-derived fragments with different cleavage boundaries. Relative abundance may use an endogenous transcript, global mean, spike-in, input volume, or particle count as denominator. Two laboratories can therefore report the same name while measuring non-equivalent quantities.

Canonical miRNAs and 3′ isomiRs may differ by one nucleotide, and evaluated poly(A)-based RT-qPCR methods cross-react between variants [46]. A sequencing signature and its RT-qPCR confirmation may, therefore, quantify different entities. Library-preparation bias adds a second source of mismatch [44].

Relative expression also depends on the selected denominator. In heterogeneous seminal plasma, an apparent change may reflect the target, reference, or both. No comprehensive cross-phenotype reference-transcript stability study was identified within the retrieved sources. An unvalidated denominator prevents threshold transportability.

**Figure 2:**
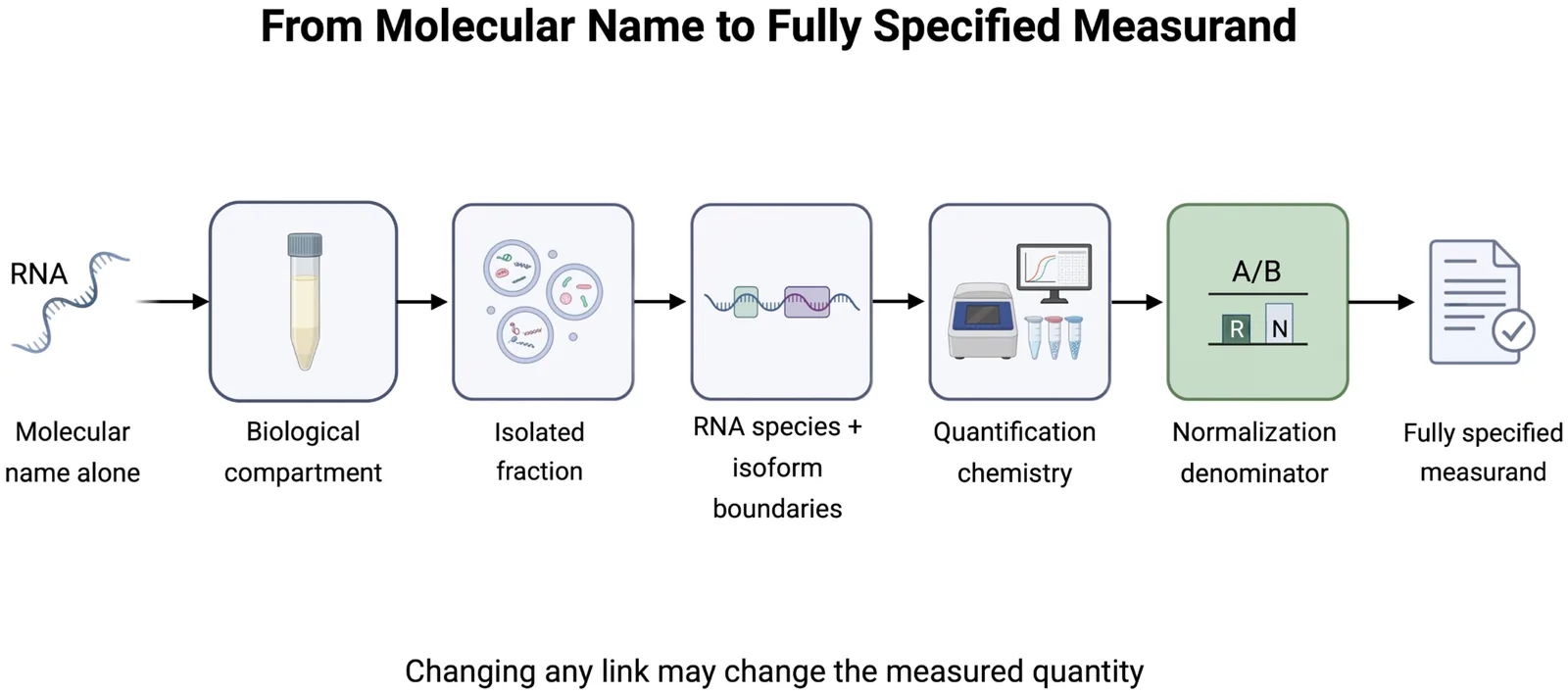
An ncRNA biomarker is a fully specified measurement chain. Transportability requires specification of the compartment, isolated fraction, RNA sequence and isoform boundaries, quantification chemistry, and normalization denominator. Changing any link may change the measured quantity. This is a conceptual model, not pooled quantitative evidence. Created in BioRender. Kaltsas, A. (2026) https://BioRender.com/r6b6tp7, (accessed 16 August 2026).

MIQE and MISEV2023 provide relevant reporting guidance [31,32]. Because no item-level audit was performed, adherence was not scored. Figure 2 depicts the measurand as a chain of specifications whose alteration can change the measured quantity.

### 6.3. Clinical Validity and Model Performance

Discrimination is necessary but insufficient. Calibration determines whether a predicted probability can be quoted credibly to a couple [103]. Precision, threshold transportability, and decision-analytic performance are also necessary.

Model sample size depends on outcome prevalence, candidate predictors, and target shrinkage; external validation has separate precision requirements [104,105]. Small high-dimensional case–control studies are prone to optimism, making independent validation essential [106,107].

Incremental value must be tested in the same patients. The nine-lncRNA panel outperformed reduced clinical and hormone models [59] but was not added to a locked contemporary model, and calibration with threshold-specific net benefit was not reported. No universal ΔAUC justifies an ncRNA assay: value depends on calibration, net benefit, assay failure, turnaround, cost, and consequences of false results. A credible study should compare current care alone with the identical model plus a locked ncRNA result [108,109].

Recent non-molecular models define a heterogeneous benchmark. Xi et al. reported a publication-described external-cohort AUC of 0.8301 in a multicenter preoperative model of more than 2800 men [92]. An older externally validated routine-variable model had an AUC of 0.65 [93]. A testosterone-to-FSH ratio produced AUCs of 0.81 in derivation and 0.74 in an independent multi-institutional validation cohort, although each sample included only 30 men [94]. Other reports include a 3093-person multicenter development nomogram [95], a six-center model that included final histopathology [96], and a single-center testicular sperm aspiration (TESA) model without independent validation [97]. Different procedures, case mixes, and predictor timings preclude cross-study AUC ranking or inference of ncRNA added value.

The appropriate evidentiary threshold depends on the use. OA/NOA classification requires performance in consecutive unresolved cases against blinded composite adjudication. Retrieval counseling requires calibrated probabilities under a standardized procedure and special protection against harmful false negatives. ART prediction requires couple- and cycle-level analysis with patient-important outcomes. Monitoring additionally requires longitudinal repeatability, responsiveness, and proof that serial results alter management. The five-domain framework organizes these questions but does not make them identical.

Calibration should be reported graphically and numerically, including calibration in the large and calibration slope when appropriate. Decision curves should compare clinically relevant strategies across plausible thresholds. Reclassification measures require prespecified risk categories linked to management. A detectable ΔAUC without improved calibration or net benefit may not justify added sampling, cost, and analytical complexity.

These clinical models do not establish a single standard-of-care calculator. Some use routine preoperative variables, others include histopathology or a different retrieval procedure, and several rely on small or same-center evaluations. They nevertheless show that credible comparators can already attain moderate discrimination and, in selected studies, external evaluation. An ncRNA panel should therefore be tested against the strongest feasible preoperative model for the same population rather than against one hormone or an unrelated cohort estimate.

The Standards for Reporting Diagnostic Accuracy Studies (STARD) and the Transparent Reporting of a multivariable prediction model for Individual Prognosis Or Diagnosis + Artificial Intelligence (TRIPOD+AI) guide reporting [110,111]. QUADAS-3 and PROBAST + AI address design-specific risk of bias and applicability [29,30]. These instruments should not be collapsed into a common score. Supplementary Table S1, therefore, reports non-scored descriptors. Extreme-group sampling, retrospective banks, and outcome-informed analysis remain common routes to optimistic performance [112].

### 6.4. Clinical Utility and Implementation

Clinical utility requires evidence that testing changes a decision and improves patient-important outcomes. No completed prospective decision-impact or randomized ncRNA-guided study was identified within the retrieved publications and registries. Proposed cutoffs and decision curves are model-evaluation evidence, not demonstrated utility [113,114].

Randomization is not required for every early evaluation, but management consequences must be specified before testing. A diagnostic classifier might reduce invasive assessment or delay, whereas a prognostic score might change counseling without changing surgery. A predictive marker requires evidence of differential treatment benefit. For each pathway, the comparator, threshold, downstream action, false-result consequences, and patient-important endpoint should be prespecified.

Implementation requires routine-laboratory reproducibility, turnaround and failure criteria, quality assurance, cost, and regulatory planning. In Europe, an assigned medical-purpose assay falls within Regulation (EU) 2017/746 on in vitro diagnostic medical devices; commercial and health-institution pathways have distinct evidence and quality requirements [115,116]. This brief regulatory context is included only because delivery constraints can prevent otherwise promising biomarkers from reaching care [117].

### 6.5. Target-Product Profile

A target-product profile for retrieval counseling should specify consecutive men with confirmed NOA before the first micro-TESE. It should define key exclusions, a locked specimen and RNA identity, assay and normalization rules, and a standardized surgical and laboratory endpoint. Validation should compare current care with and without ncRNA; evaluate calibration, positive predictive value (PPV), negative predictive value (NPV), and net benefit across intended-use prevalence and thresholds; and carry statistical uncertainty through the analysis. Results should support shared decisions rather than automatic denial of surgery.

The endpoint should distinguish any observed sperm from sperm adequate for cryopreservation or ICSI. Turnaround, assay failure, repeat sampling, cost, external quality assessment, and geographic transportability should also be prespecified. PPV and NPV must be estimated within the intended prevalence rather than calculated from optimistic internal point estimates. This is why the submitted hypothetical NPV example was removed.

### 6.6. Ethical and Counseling Considerations

Exclusionary use has the highest evidentiary threshold because a false negative could remove a couple’s only route to genetic parenthood. External validation, calibration, threshold-specific net benefit, impact evidence, and stakeholder-accepted residual risk are all required. Counseling should remain couple-centered [118].

## 7. Molecule-Independent Challenges to Validation and Synthesis

Outcome validity is as important as assay validity: retrieval is protocol-dependent, and diagnostic references can be spatially heterogeneous or imperfect.

### 7.1. A Protocol-Dependent Retrieval Endpoint

Retrieval is often treated as a fixed binary attribute, although sampling and laboratory search affect observation. In 93 men undergoing sequential micro-TESE and trifocal testicular sperm extraction (TESE), sperm was found in 58.1% and 54.8% of separate specimens, while combined assessment yielded 63.4% [119]. The retrospective sequence supports protocol conditionality, not superiority of either technique.

Operation type, laterality, sampling, search duration, and the definition of success can, therefore, affect apparent external performance. Studies should standardize or model these factors and distinguish empirical hormonal optimization from treatment of hypogonadotropic hypogonadism [120,121].

A center using another operation, a different tissue-sampling strategy, or a longer embryology search can assign a different observed outcome to a similar patient. Prior hormonal treatment adds a center-dependent co-intervention, and evidence for routine use in primary spermatogenic failure remains limited [120]. Hypogonadotropic hypogonadism is different: spermatogenesis may recover after gonadotropin therapy, so untreated or incompletely treated patients should not be mixed with irreversible primary testicular failure [121].

Outcome reporting should identify the analysis unit and classification timing. Bilateral testes, multiple fragments, repeated searches, and salvage procedures create correlated observations within one patient. Models should retain the patient as the clinical unit unless a justified hierarchy is specified. External validation should preserve the intended operation and counseling point or quantify how protocol differences modify calibration.

### 7.2. An Imperfect and Spatially Heterogeneous Reference Standard

OA/NOA assignment is a composite clinical judgment [1,2,4]. Histopathology can inform endotyping but samples a heterogeneous organ. In one NOA cohort, diagnostic biopsy missed sperm later found at therapeutic retrieval in 11 of 22 retrieval-positive men [122]. In another series, 28 of 104 men with bilateral samples had discordant histological patterns, and eight showed mature sperm on only one side [123]. These single-center findings demonstrate sampling heterogeneity, not universal error rates.

Near-perfect accuracy against selected groups should, therefore, prompt scrutiny of spectrum and reference assignment. Options include blinded composite adjudication, sensitivity analyses, and defensible latent-class methods when no single gold standard exists [124].

Reference misclassification can inflate or attenuate an association, and its direction cannot be inferred from bilateral-biopsy data alone. Histology should not be treated as an infallible binary gold standard when focal spermatogenesis is possible. A composite standard should define the contributing clinical, hormonal, genetic, operative, and pathological information, as well as the adjudicators, masking, and handling of mixed or equivocal cases.

**Figure 3:**
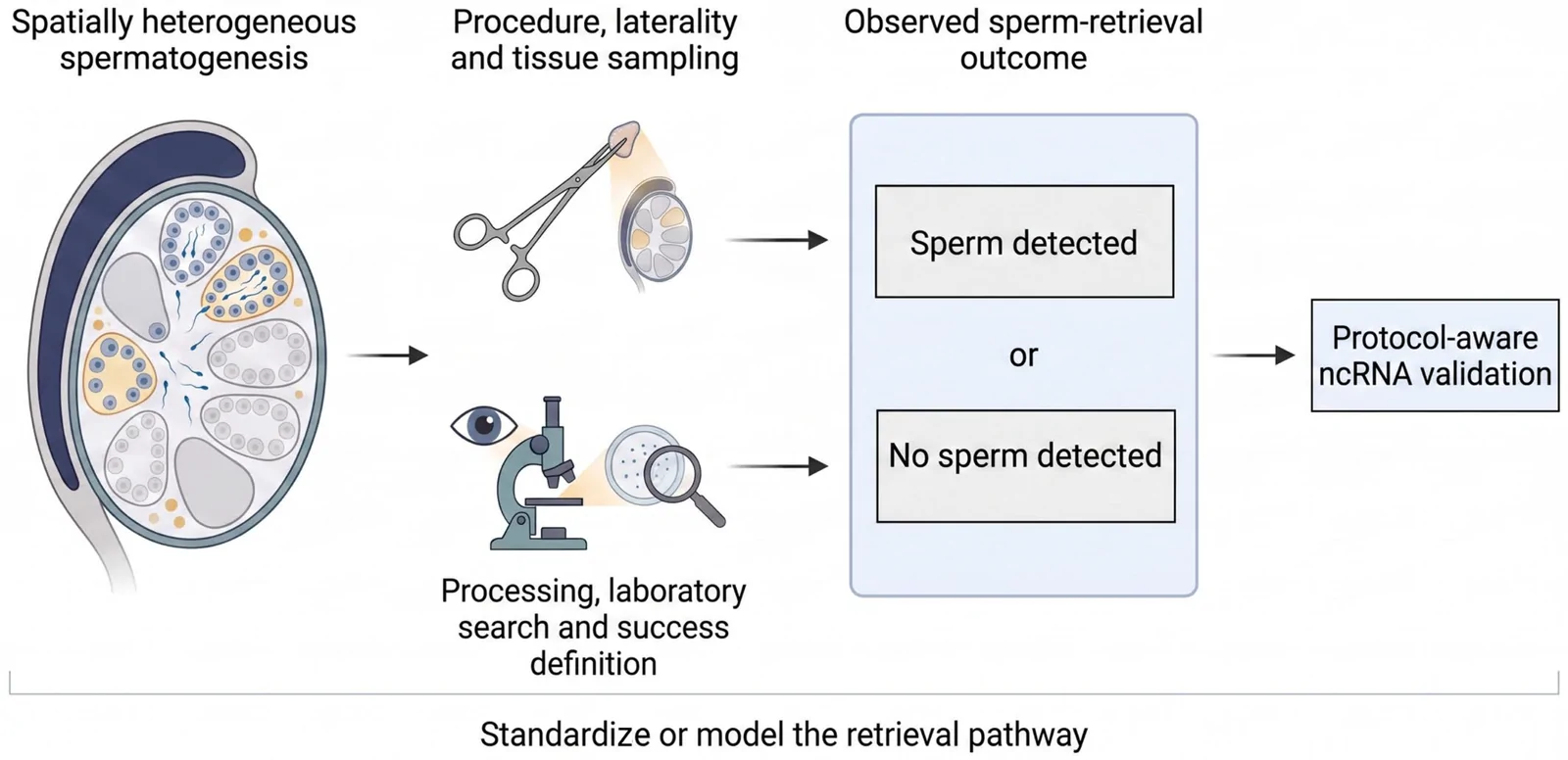
Protocol-defined sperm-retrieval outcomes in a spatially heterogeneous testis. Heterogeneous spermatogenesis, surgical sampling, laboratory processing, search, and the definition of success jointly condition the observed result. The recorded outcome is, therefore, a protocol-defined observation rather than an entirely fixed patient attribute. This conceptual model does not imply the superiority of a technique, a universal error rate, or proof that a negative observation means complete absence of sperm. Created in BioRender. Kaltsas, A. (2026) https://BioRender.com/p3zbdex, (accessed 16 August 2026).

OA/NOA remains clinically useful because it maps to management, not because all patients occupy perfectly separable biological states. Partial obstruction and mixed pathology define the boundary where a test might add most value and where adjudication must be clearest. Figure 3 integrates tissue heterogeneity with the retrieval protocol.

### 7.3. Limits of Quantitative Synthesis

Quantitative synthesis requires commensurate measurands and comparable reference or procedural endpoints. Otherwise, pooling can produce a precise estimate for an inadequately defined target. Cohort overlap adds a second hazard: the EV program [49,50,51] contains confirmed reuse, and the 2026 plasma studies [64,65,66] report the same cohort. These reports must not be counted as independent replications.

The overlap assessment therefore compared authorship, center, recruitment period, sample size, phenotype distribution, specimen, and validation role across the selected reports. Independence was marked confirmed only when the publication documented a distinct cohort. Shared authorship alone was not treated as overlap, and missing provenance was not converted into a claim of duplication. Supplementary Tables S1 and S2 record the report- and program-level judgments.

This distinction affects synthesis directly. Pooling several reports from one specimen bank would narrow uncertainty without adding independent information. Conversely, excluding every report with shared authors could discard legitimate temporal or geographic replication. Program-level grouping is, therefore, a provisional evidence-unit decision. It should be updated if authors provide participant identifiers, recruitment dates, or explicit statements confirming unique cohorts.

### 7.4. Implications for Multi-Omics

Multi-omic models may combine ncRNAs with proteins, exome findings, or transcriptomic context [5,6,35,36,37,70], but they do not repair an unstable outcome. Panels and preprocessing must be locked before external evaluation, while reference adjudication and procedural endpoints are improved in parallel.

Flexible integration increases the opportunity to fit center-specific patterns in specimen handling, pathology, and outcome definition. Internal accuracy can improve while transportability worsens. Multi-omic development should therefore use prespecified clinical roles for each data layer, prevent leakage across feature selection and validation, and retain a clinically usable comparator model. Complexity is justified only when it adds reproducible decision value. Against this background, Table 3 summarizes, for each of the six intended uses, the current evidence maturity and the minimum unmet requirement before clinical use.

## 8. Evidence Gaps and Research Roadmap

Six priorities form a dependency chain. First, define the intended population, comparator, reference, and management consequence. Second, lock the compartment, fraction, sequence, assay, and denominator, with MIQE and MISEV2023 reporting where applicable [31,32,41,46]. Third, standardize the retrieval procedure, laboratory search, and success definition [119].

The first three priorities determine whether studies are measuring the same test against the same target. Fertile and normozoospermic controls should be separated, mixed OA/NOA cases should be adjudicated explicitly, and the intended point in the pathway should be declared. Specimen processing should include contamination checks and interlaboratory reproducibility. Retrieval studies should report laterality, tissue sampling, search duration, and whether success means any sperm or usable sperm.

Fourth, use prospective designs, prespecified thresholds, and samples sized for calibration as well as discrimination [104,105]. Fifth, undertake temporal and geographic validation and compare current care with and without ncRNA in the same patients [106,107,108]. Sixth, evaluate decision impact, implementation, cost, and couple-centered outcomes only after the preceding foundations are secure [84,113,114].

A registered analysis plan should separate discovery, model development, and locked evaluation. Validation should report calibration in the large, calibration slope, discrimination with confidence intervals, and decision curves over clinically plausible thresholds. Utility studies should measure how results change counseling or treatment and whether those changes improve outcomes. Couple-level endpoints and treatment burden should accompany laboratory outcomes.

## 9. Limitations of This Review

This review used purposive selection and one-reviewer screening. PubMed was the only database with a fully documented reproducible search; archived Embase and Scopus exports could support only a retrospective coverage audit because exact query-version provenance was uncertain. Gray literature and non-English reports were not sought systematically. The submitted PubMed query contained an invalid tRF truncation, although sensitivity testing found no missed decision-relevant human tRF report. No formal duplicate risk-of-bias assessment or quantitative synthesis was performed. Absence statements are bounded search findings and remain less secure than conclusions from exhaustive systematic retrieval.

The selected 23 decision-relevant or contextual reports in Supplementary Table S1 are an auditable analytic subset, not an included-study denominator. The coverage audit confirms that most of this subset appeared in each archived export, but it cannot quantify studies omitted before selection. Database indexing, publication timing, non-English literature, and unavailable cohort details may therefore affect both evidence mapping and program-overlap judgments.

The maturity categories also depend on published reporting. An apparently separate sample may share participants, specimen banks, preprocessing, or feature selection that the report does not reveal. Conversely, shared investigators do not prove participant overlap. These uncertainties were retained rather than resolved by assumption.

The protocol and tissue-heterogeneity examples were retrospective, single-center studies and do not estimate universal error rates [119,122,123]. The 31 August update identified one new abstract-level circRNA/miRNA report from the same broad research program [72]. Because the accessible abstract did not report enough cohort provenance to establish independence, it was tabulated as an update and not treated as external validation. The review does not appraise ncRNA therapeutics or broader mechanistic biology.

## 10. Conclusions

Evidence is most mature for OA/NOA classification and retrieval prognosis, but it remains dominated by selected case–control studies, single-center development, and same-program evaluation. Within the reported searches, no independent geographic validation of a locked ncRNA assay, complete nested comparison with contemporary preoperative care, or prospective study of ncRNA-guided management was identified. Current guidelines do not recommend routine ncRNA testing; other intended uses remain exploratory.

Translation depends on fully specified measurands, representative populations, credible reference standards, standardized retrieval endpoints, calibrated same-patient added value, and evidence of benefit of relevant decisions. Meeting these design requirements, rather than expanding discovery alone, will determine whether ncRNA biology becomes clinically actionable.

## Figures and Tables

**Table 1:** Non-coding RNA classes relevant to male reproduction: biological or mechanistic relevance, human clinical biomarker evidence, and principal measurement issues.

Class	Biological or Mechanistic Relevance	Human Clinical Biomarker Evidence	Principal Measurement Issue
miRNA	Post-transcriptional repression across spermatogenesis [22].	Most studied class; human reports address infertility detection, OA/NOA classification, retrieval, and ART associations [47,48,49,50,51,52,53].	Closely related isomiRs may cross-react in some poly(A)-based RT-qPCR assays [46].
piRNA	PIWI-dependent transposon repression and germline integrity [9,10].	Seminal-plasma associations and small internal retrieval models have been reported [47,54].	Short-read annotations vary and can overlap tRNA-fragment assignments.
tsRNA/tRF	Animal experiments support roles in intergenerational signaling but do not establish human causality [33,34].	Human studies report OA/NOA, retrieval, and ART associations; independent validation is absent [55,56,57,58].	Base modifications can impede adaptor ligation and reverse transcription [45].
lncRNA	Chromatin and transcript regulation during germ-cell development [11].	A nine-lncRNA retrieval panel has strong internal but no geographic validation [59]; other evidence is exploratory [60].	Low abundance, complex isoforms, and normalization can limit transferability.
circRNA	Regulatory scaffolding and transcript interactions; embryo evidence includes a bovine model [12,16].	Several single-center retrieval models report high apparent discrimination [61,62,63,64,65,66].	Back-splice junctions require dedicated pipelines and exact isoform specification.
rRNA- and Y-RNA-derived fragments	Abundant in some biofluids; functions remain incompletely resolved [20].	Sperm 28S rRNA fragments have been associated with embryo quality, without a locked validated test [67].	Standard pipelines may discard these biotypes; reporting should remain separate.

Note: circRNA, circular RNA; EV, extracellular vesicle; lncRNA, long non-coding RNA; miRNA, microRNA; piRNA, PIWI-interacting RNA; RT-qPCR, reverse transcription quantitative polymerase chain reaction; rRNA, ribosomal RNA; tRF, tRNA-derived fragment; tsRNA, tRNA-derived small RNA. tsRNA/tRF nomenclature is not fully standardized; sequence, length, cleavage origin, and annotation database should be reported.

**Table 2:** Selected human evidence by intended use, with explicit design and cohort-independence descriptors. Direct ncRNA evidence is separated from contextual clinical or mixed-RNA evidence.

Intended Use/Evidence Role	Analyte and Specimen	Design and Setting	Cohort and Reported Result	Validation, Independence, and Principal Limitation
Direct ncRNA—infertility detection	Five miRNAs; sperm/testis [48]	Clinic-based case–control; timing NR; single program	226 men; individual AUCs of 0.78–0.99; CIs NR	Development; no separate cohort; extreme groups and no combined-panel result
Direct ncRNA—infertility detection	Five piRNAs; seminal plasma [47]	Case–control association; timing NR; single center/program	211 infertile and 91 fertile men; no decision AUC	Development; no independent cohort or decision threshold
Direct ncRNA—OA/NOA	miR-31-5p; seminal plasma and EV-enriched fractions [50]	Prospective case–control follow-up; same program	Selected OA and secretory/cryptozoospermia groups; AUC of 0.72–0.88 overall	Same program; independence not established; vasectomy-dominated OA spectrum
Direct ncRNA—OA/NOA	Plasma EV miR-202-5p/miR-513c-5p plus FSH [52]	Single-center selected-group model; timing NR	Selected OA and NOA groups; ROC performance reported	Development; no independent external cohort or nested clinical comparator
Direct ncRNA—retrieval	Nine lncRNAs; seminal-plasma EV-enriched preparation [59]	Single-center development with random holdout	Development, 30; holdout, 66; AUC, 0.99 and 0.96	Internal split; same program; no geographic validation or calibration
Direct ncRNA—retrieval	piR-61927; seminal-plasma EV-enriched preparation [54]	Single-center training and internal evaluation	20 and 25 men; AUC of 0.82 and 0.83	Internal; independent cohort not established; wide CIs
Direct ncRNA—retrieval	Three circRNAs; seminal plasma [61]	Single-program discovery and model development	Discovery, 6; model cohort, 52; combined AUC, 0.96	Apparent performance; the 52-person cohort was reused
Direct ncRNA—retrieval	Four miRNAs; seminal plasma [53]	Single-center screening, training, and blinded internal test	18, 56, and 40 specimens; test AUC of 0.93	Internal split; no geographic validation or calibration
Direct ncRNA—retrieval	Two plasma EV tRFs, described as exosomal in the source [56]	Small selected case–control study	12 SR+, 18 SR−, and 12 fertile; AUCs of 0.92 and 0.95	Development; no independent cohort or clinical comparator
Direct ncRNA—OA/NOA and retrieval	tRF-Val-AAC-010; seminal-plasma EV-enriched preparation [55]	Two-stage single-program study	18 SR+, 23 SR− plus comparators; retrieval AUC of 0.89	Development; origin AUC of 0.96 must not be transferred to retrieval
Direct ncRNA—retrieval	Six circRNAs; serum [62]	Single-center model; screening subset reused	20 of 180 screened; full cohort of 84 SR+/96 SR−; AUC of 0.98	Apparent performance; no clean internal or geographic validation
Direct ncRNA—retrieval	Plasma RNA axes [64,65,66,72]	Four 2026 reports from one investigative program	Refs. [64,65,66] used the same 60-NOA/40-control cohort; Ref. [72] cohort NR; AUCs of 0.909–0.983	One confirmed cohort for Refs. [64,65,66]; Ref. [72] participant independence unresolved; no independent replication
Direct ncRNA—retrieval association	circ_MGLL; testicular tissue obtained during micro-TESE [63]	Retrospective single-center split sample	114 men; training, 58/validation, 56; AUC 0.868/0.811	Internal; predictors unavailable preoperatively; no geographic validation
Contextual current-care models	Routine clinical variables, with histopathology in some models [92,93,94,95,96,97]	Retrospective/prospective, single- and multicenter models	Cohorts 333 to >3000; reported AUC/C-index of about 0.65–0.84	Some external validation; predictor timing and procedures differ; no head-to-head ncRNA comparison
Contextual mixed-RNA evidence	Predominantly exonic sperm RNA elements [77]	Retrospective couple/pathway association	96 enrolled; 72 passed QC; signature defined in seven live-birth controls	Development; no independent replication; not ncRNA-specific
Direct ncRNA—ART	Sperm 28S rRNA fragments [67]	Retrospective single-center cohort	135 IVF couples; association with embryo quality	Development; intermediate endpoint; no independent cohort
Direct ncRNA—ART	Selected- and bulk-sperm miRNAs [83]	13-donor discovery with same-program bulk-sperm follow-up	39 nested libraries plus 85 men; combined AUC of 0.75	Same-program; measurand changed; no external validation
Direct ncRNA—ART	Sperm small-noncoding-RNA profile [81]	Prospective recruitment; high-dimensional group comparison	54 sequenced; selected groups of 18/14/12; no locked classifier	Discovery; intermediate endpoint and no independent validation

Note: Performance estimates are reproduced as reported and should not be ranked across different populations, procedures, prevalence, references, or validation designs. “Independent” requires documented temporal or geographic separation; unresolved overlap is labeled explicitly. Abbreviations: ART, assisted reproductive technology; AUC, area under the receiver operating characteristic curve; CI, confidence interval; EV, extracellular vesicle; FSH, follicle-stimulating hormone; NOA, non-obstructive azoospermia; NR, not reported; OA, obstructive azoospermia; QC, quality control; ROC, receiver operating characteristic; SR+/SR−, successful/failed sperm retrieval; tRF, tRNA-derived fragment.

**Table 3:** Use-specific clinical-actionability matrix. Evidence maturity uses the descriptive criteria defined in Section 2.5 and is not a numerical score.

Intended Use	Decision Informed	Current Comparator	Evidence Maturity	Minimum Unmet Requirement
Infertility subtyping	Management after an otherwise non-diagnostic evaluation	Complete couple and andrological evaluation	Discovery associations	Representative clinical cohort; predefined molecular target and management consequence
OA versus NOA	OA-directed treatment versus NOA counseling and retrieval planning	Full guideline-based work-up [1,2,3,4]	Same-program selected-group evaluation [50]	Consecutive equivocal cases; blinded composite adjudication; decision-impact analysis
Retrieval prognosis in NOA	Calibrated counseling before first micro-TESE	Contemporary clinical models and integrated assessment [92,93,94,95,96,97]	Development and internal evaluation; no independent geographic ncRNA validation	Locked preoperative assay; standardized retrieval endpoint; same-patient added value, calibration, and net benefit
ART outcome prediction	Whether a male result should alter ART strategy	Female age, ovarian response, embryo and laboratory factors	Exploratory, mainly intermediate outcomes	Couple/cycle analysis; external validation; cumulative live birth or other patient-important outcome
Beyond-semen assessment	Management when conventional semen parameters are unremarkable	Complete couple evaluation; no single molecular reference	Sparse direct ncRNA evidence; contextual mixed-RNA program [77]	Incremental value beyond complete evaluation against reproductive outcomes
Monitoring	Whether serial change should alter treatment	Clinical review and serial semen analysis	Hypothesis-generating	Within-person repeatability; minimal-change threshold; responsiveness; evidence that serial testing improves management

Note: Discovery denotes association or apparent performance without a separate evaluation sample. Internal validation denotes split-sample, resampling, or same-center evaluation. Same-program validation denotes follow-up without established program independence. External validation requires a locked assay or model in an independent temporal or geographic population. Clinical utility requires improved decisions, net benefit, or patient-important outcomes. Abbreviations: ART, assisted reproductive technology; micro-TESE, microdissection testicular sperm extraction; ncRNA, non-coding RNA; NOA, non-obstructive azoospermia; OA, obstructive azoospermia.

## Data Availability

No new primary data were created. Structured review-level descriptors are provided in Supplementary Table S1.

## Supplementary Materials

The following supporting information can be downloaded at: https://www.mdpi.com/article/10.3390/genes17091074/s1, Table S1: Report-level design, validation, and independence descriptors; Table S2: Cohort-overlap and evidence-unit map.

## Abbreviations

The following abbreviations are used in this manuscript:

ART	Assisted reproductive technology
AUA/ASRM	American Urological Association/American Society for Reproductive Medicine
AUC	Area under the receiver operating characteristic curve
AZF	Azoospermia factor
CI	Confidence interval
circRNA	Circular RNA
EAU	European Association of Urology
ECM1	Extracellular matrix protein 1
EV	Extracellular vesicle
FDA–NIH BEST	Food and Drug Administration–National Institutes of Health Biomarkers, EndpointS, and other Tools
FSH	Follicle-stimulating hormone
ICSI	Intracytoplasmic sperm injection
ICTRP	International Clinical Trials Registry Platform
IUI	Intrauterine insemination
IVF	In vitro fertilization
lncRNA	Long non-coding RNA
micro-TESE	Microdissection testicular sperm extraction
MIQE	Minimum Information for Publication of Quantitative Real-Time PCR Experiments
miRNA	MicroRNA
MISEV2023	Minimal Information for Studies of Extracellular Vesicles
ncRNA	Non-coding RNA
NOA	Non-obstructive azoospermia
NPV	Negative predictive value
NR	Not reported
OA	Obstructive azoospermia
PANDORA-seq	Panoramic RNA display by overcoming RNA modification aborted sequencing
piRNA	PIWI-interacting RNA
PPV	Positive predictive value
PROBAST+AI	Prediction model Risk Of Bias ASsessment Tool + Artificial Intelligence
QC	Quality control
QUADAS-3	Quality Assessment of Diagnostic Accuracy Studies, version 3
ROC	Receiver operating characteristic
rRNA	Ribosomal RNA
RT-qPCR	Reverse transcription quantitative polymerase chain reaction
SANRA	Scale for the Assessment of Narrative Review Articles
SR+/SR−	Successful/failed sperm retrieval
STARD	Standards for Reporting Diagnostic Accuracy Studies
TESA	Testicular sperm aspiration
TESE	Testicular sperm extraction
TEX101	Testis-expressed protein 101
tRF	tRNA-derived fragment
TRIPOD + AI	Transparent Reporting of a multivariable prediction model for Individual Prognosis Or Diagnosis + Artificial Intelligence
tsRNA	tRNA-derived small RNA
WHO	World Health Organization

## Appendix A. PubMed/MEDLINE Search and Sensitivity Audit

Search executed on 13 August 2026; database inception to 13 August 2026; 883 records returned. Exact query:

((“Infertility, Male”[Mesh] OR “male infertility”[tiab] OR “male subfertility”[tiab] OR azoosperm*[tiab] OR oligozoosperm*[tiab] OR asthenozoosperm*[tiab] OR teratozoosperm*[tiab] OR spermatozo*[tiab] OR “sperm retrieval”[tiab] OR “testicular sperm”[tiab] OR micro-TESE[tiab] OR microTESE[tiab] OR varicocele[tiab] OR “recurrent pregnancy loss”[tiab]) AND (“RNA, Untranslated”[Mesh] OR “MicroRNAs”[Mesh] OR “RNA, Long Noncoding”[Mesh] OR “noncoding RNA”[tiab] OR “non-coding RNA”[tiab] OR ncRNA*[tiab] OR “small noncoding RNA”[tiab] OR “small non-coding RNA”[tiab] OR sncRNA*[tiab] OR microRNA*[tiab] OR miRNA*[tiab] OR isomiR*[tiab] OR piRNA*[tiab] OR tsRNA*[tiab] OR tRF*[tiab] OR “tRNA-derived”[tiab] OR “transfer RNA-derived”[tiab] OR “rRNA-derived”[tiab] OR “ribosomal RNA fragment”[tiab] OR YRNA*[tiab] OR “Y RNA”[tiab] OR lncRNA*[tiab] OR “long noncoding RNA”[tiab] OR “long non-coding RNA”[tiab] OR circRNA*[tiab] OR “circular RNA”[tiab] OR “small RNA”[tiab] OR “sperm RNA”[tiab] OR “extracellular vesicle RNA”[tiab]) AND (biomarker*[tiab] OR diagnos*[tiab] OR prognos*[tiab] OR predict*[tiab] OR “sperm retrieval”[tiab] OR “testicular sperm”[tiab] OR fertilization[tiab] OR fertilisation[tiab] OR embryo*[tiab] OR pregnancy[tiab] OR “live birth”[tiab] OR “assisted reproduction”[tiab] OR IVF[tiab] OR ICSI[tiab] OR monitor*[tiab] OR treatment[tiab])) AND (“1900/01/01”[Date—Publication]: “2026/08/13”[Date—Publication])

Methodological note: The executed string is preserved verbatim. PubMed requires four characters before a truncation wildcard, so tRF*[tiab] did not expand. The same block nevertheless contained tsRNA*[tiab], “tRNA-derived”[tiab], and “transfer RNA-derived”[tiab]. The structured strategy was specificity-oriented and was not treated as exhaustive.

### Appendix A.1. tRF/tsRNA Sensitivity Assessment

Assessment executed on 31 August 2026 with the original publication-date cap. The original query returned 886 records after dynamic indexing. Replacing only tRF*[tiab] with (tRF[tiab] OR tRFs[tiab]) returned 887; the single additional record was a mouse Trf2 study unrelated to tRNA-derived fragments or clinical male-infertility biomarkers. The corrected structured-query subset defined by tRF[tiab], tRFs[tiab], tsRNA*[tiab], “tRNA-derived”[tiab], or “transfer RNA-derived”[tiab] returned 35 records.

Corrected sensitivity query (exact string):

((“Infertility, Male”[Mesh] OR “male infertility”[tiab] OR “male subfertility”[tiab] OR azoosperm*[tiab] OR oligozoosperm*[tiab] OR asthenozoosperm*[tiab] OR teratozoosperm*[tiab] OR spermatozo*[tiab] OR “sperm retrieval”[tiab] OR “testicular sperm”[tiab] OR micro-TESE[tiab] OR microTESE[tiab] OR varicocele[tiab] OR “recurrent pregnancy loss”[tiab]) AND (“RNA, Untranslated”[Mesh] OR “MicroRNAs”[Mesh] OR “RNA, Long Noncoding”[Mesh] OR “noncoding RNA”[tiab] OR “non-coding RNA”[tiab] OR ncRNA*[tiab] OR “small noncoding RNA”[tiab] OR “small non-coding RNA”[tiab] OR sncRNA*[tiab] OR microRNA*[tiab] OR miRNA*[tiab] OR isomiR*[tiab] OR piRNA*[tiab] OR tsRNA*[tiab] OR (tRF[tiab] OR tRFs[tiab]) OR “tRNA-derived”[tiab] OR “transfer RNA-derived”[tiab] OR “rRNA-derived”[tiab] OR “ribosomal RNA fragment”[tiab] OR YRNA*[tiab] OR “Y RNA”[tiab] OR lncRNA*[tiab] OR “long noncoding RNA”[tiab] OR “long non-coding RNA”[tiab] OR circRNA*[tiab] OR “circular RNA”[tiab] OR “small RNA”[tiab] OR “sperm RNA”[tiab] OR “extracellular vesicle RNA”[tiab]) AND (biomarker*[tiab] OR diagnos*[tiab] OR prognos*[tiab] OR predict*[tiab] OR “sperm retrieval”[tiab] OR “testicular sperm”[tiab] OR fertilization[tiab] OR fertilisation[tiab] OR embryo*[tiab] OR pregnancy[tiab] OR “live birth”[tiab] OR “assisted reproduction”[tiab] OR IVF[tiab] OR ICSI[tiab] OR monitor*[tiab] OR treatment[tiab])) AND (“1900/01/01”[Date—Publication]: “2026/08/13”[Date—Publication])

Targeted follow-up query (exact string; 51 records):

((“Infertility, Male”[Mesh] OR “male infertility”[tiab] OR “male subfertility”[tiab] OR azoosperm*[tiab] OR oligozoosperm*[tiab] OR asthenozoosperm*[tiab] OR teratozoosperm*[tiab] OR spermatozo*[tiab] OR “sperm retrieval”[tiab] OR “testicular sperm”[tiab] OR micro-TESE[tiab] OR microTESE[tiab] OR varicocele[tiab] OR “recurrent pregnancy loss”[tiab]) AND (tRF[tiab] OR tRFs[tiab] OR tsRNA*[tiab] OR “tRNA-derived”[tiab] OR “transfer RNA-derived”[tiab] OR “extracellular RNA”[tiab] OR “exosomal RNA”[tiab] OR “sperm-borne RNA”[tiab])) AND (“1900/01/01”[Date—Publication]: “2026/08/13”[Date—Publication])

The targeted query yielded 17 records absent from the submitted query. Title screening found no additional decision-relevant human study. All four human tRF reports discussed in the review (PMIDs 33693947, 40890583, 35869479, and 34955241) were retrieved by both the submitted and corrected structured queries.

### Appendix A.2. Update Through 31 August 2026

The corrected query with the publication end date changed to 31 August 2026 returned 890 records. Three records postdated the submitted search: one lncRNA-polymorphism meta-analysis outside the direct biomarker scope, one mouse treatment study, and one new human circRNA/miRNA retrieval report [72]. The last was recorded as an abstract-level update because cohort independence could not be established from the accessible abstract.

### Appendix A.3. Archived Export Coverage Audit

Archived 24 July 2026 exports contained 1967 PubMed, 3038 Embase, and 2587 Scopus records. Exact query-version provenance for the Embase and Scopus exports could not be reconstructed with certainty; they were therefore not presented as formal reproducible searches. DOI matching located 22, 21, and 19 of the 23 reports in the submitted Supplementary Table S1, respectively. The unmatched reports largely postdated the exports. This check assessed coverage of a selected evidence set and did not constitute complete screening of the exported records.

## Appendix B. Trial-Registry Searches

Searches were executed on 13 August 2026 without restriction by recruitment status. Counts refer to records returned by each platform’s search engine; registrations without posted results were treated as ongoing evidence rather than completed validation.

ClinicalTrials.gov—6 records. Exact query: (“non-coding RNA” OR microRNA OR lncRNA OR circRNA OR piRNA OR “tRNA-derived”) AND (“male infertility” OR azoospermia OR “sperm retrieval”). The most directly retrieval-related record was NCT02932865, a prospective observational study marked completed without posted registry results; none of the six records tested ncRNA-guided management.

ISRCTN—0 records. Exact query: (microRNA OR miRNA OR lncRNA OR circRNA OR piRNA OR noncoding OR ncRNA OR “tRNA-derived”) AND (“male infertility” OR azoospermia OR “sperm retrieval”).

WHO ICTRP—3 records for 3 trials. Exact query: (microRNA OR miRNA OR lncRNA OR circRNA OR piRNA OR ncRNA OR “non-coding RNA” OR “tRNA-derived”) AND (“male infertility” OR azoospermia OR “sperm retrieval”). One record was directly relevant: ChiCTR2100046379, a prospective circRNA retrieval-prediction study targeting 200 participants, listed as recruiting without posted results. The other two records concerned genome-variant identification and yoga, respectively.
