# Supplementary material for: Non-Coding RNA Biomarkers in Male Infertility: From Discovery to Clinical Actionability—A Narrative Review
Source: Genes (Basel). 2026 Sep 6;17(9):1074. doi: 10.3390/genes17091074 (PMC13606387; doi:10.3390/genes17091074)
Supplement: Supplementary file 1 [file genes-17-01074-s001.zip › genes-4537689-supplementary.pdf]

Supplementary Evidence Tables

Non-Coding RNA Biomarkers in Male Infertility: From Discovery to Clinical Actionability—A Narrative Review

Table S1 separates report-level evidence from cohort-level independence. It expands the submitted descriptors with design timing, sampling frame, center count, recruitment period, selection timing, validation stage, outcome level, and overlap status. Entries marked NR were not reported in the accessible primary source. The descriptors support transparent narrative appraisal and are not a formal duplicate risk-of-bias assessment.

Table S1. Report-level design, validation, and independence descriptors

Study / evidence role	Intended use / design timing	Sampling frame / setting	Analyte / selection	Outcome / level	Validation / performance	Cohort independence	Current-care comparison / principal limitation
Abu-Halima 2014 [Ref. 48] Direct ncRNA 10.1016/j.fertnstert.2014.07.001; PMID 25108464	Infertility detection; clinic-based case–control; diagnostic-accuracy descriptors relevant; timing NR	Frame: case–control / selected clinic groups; consecutive status NR; centers NR (research/clinical institutes mentioned; recruitment structure not explicit); recruitment NR; participants: infertility-clinic men versus fertile/control groups; spectrum not a consecutive diagnostic cohort	Five-miRNA candidates; marker-level cut-offs derived within program; no externally locked assay; selection: mixed—five candidates carried forward from prior work; thresholds/performance estimated internally	Male-infertility/semen and testicular diagnostic group classification; level: intermediate/diagnostic label; reference/endpoint: clinical infertility and semen/testis group labels	Development plus internal validation sets Independence within report: Internal only; geographic/program independence not established Performance/precision: AUC range reported; AUC CIs and calibration NR	None established; no participant overlap established with other S1 reports	Comparator: No same-patient comparison with complete clinical work-up Concern: Extreme-group sampling and multiple marker analyses can overstate deployment performance Audit note: Do not treat internal validation sets as external validation.
Hong 2016 [Ref. 47] Direct ncRNA 10.1038/srep24229; PMID 27068805	Infertility detection; case–control association/panel discovery; timing NR	Frame: case–control /selected infertility phenotypes and fertile volunteers; consecutive status NR; centers: 2 clinical recruitment centers; recruitment NR; participants: 211 infertile men with selected phenotypes versus 91 fertile controls	Five-piRNA panel; decision threshold not established; selection: post hoc/data-derived feature selection	Male-infertility/semen-phenotype classification Level: intermediate/diagnostic label; reference/endpoint: phenotype and fertility-control labels	Development plus same-program internal validation cohort Independence within report: Participant-independent validation groups, but not external to the two-center program Performance/precision: No decision AUC/CI or calibration for deployment	None established; no cross-report participant overlap established	Comparator: No full clinical comparator Concern: Association across selected groups does not define an actionable diagnostic test Audit note: Internal stage separation should not be described as geographic external validation.
Plata-Peña 2023 [Ref. 50] Direct ncRNA 10.1111/andr.13286; PMID 36057114	OA versus NOA; follow-up diagnostic-accuracy study; timing NR	Frame: case–control /selected referred azoospermia groups and controls; consecutive status NR; centers: 1 clinical recruitment center (with two collaborating research institutions); recruitment: January 2016–December 2019; participants: azoospermic men, including an FSH <10 IU/L subgroup; not a geographic replication	miR-31-5p RT-qPCR; pre-analytical source examined; threshold not externally locked; selection: prespecified candidate from [49]; internal cutoff/Youden selection	Obstructive azoospermia versus spermatogenic-failure/secretory azoospermia classification; level: intermediate/diagnostic label; reference/endpoint: clinical OA/NOA assignment	Targeted experimental validation / same-program follow-up; no external cohort Independence within report: Not an independent program; substantial participant reuse with [51]; overlap with [49] remains unclear Performance/precision: AUC >0.88; AUC CI NR; calibration NR	[49–51] Fundació Puigvert/IDIBELL program confirmed substantial participant reuse with [51]; [49]↔[50] direct reuse not proven	Comparator: Subgroup assessment but no complete nested clinical-model comparison Concern: Same-program follow-up and uncertain performance in consecutive equivocal patients Audit note: Count [49–51] at the program/evidence-unit level where cohort independence matters; do not count each report as a wholly independent cohort.
Qi 2024 [Ref. 52] Direct ncRNA 10.1093/biolre/ioae130; PMID 39216109	OA versus NOA; multivariable diagnostic model (PROBAST+AI-relevant); timing NR	Frame: case–control /selected OA and NOA groups; consecutive status NR; centers: 1 (per S1; not independently verified from accessible primary abstract); recruitment NR; participants: selected OA and NOA groups at one center	Plasma EV miR-202-5p/miR-513c-5p plus FSH; model not externally locked; selection: post hoc/data-derived	Obstructive versus non-obstructive azoospermia classification; level: intermediate/diagnostic label; reference/endpoint: clinical OA/NOA diagnosis	Development/internal only Independence within report: No external cohort established Performance/precision: ROC performance reported; AUC CI/calibration for deployment NR	Possible Xuzhou-associated author/program family with [55]/[56]; participant overlap unclear; same-group clues only; no participant reuse established	Comparator: Includes FSH, but no complete clinical model alone versus plus-ncRNA comparison Concern: Single-center selected spectrum and no independent validation Audit note: Center count relies on S1 because accessible primary material was abstract-only.
Xie 2020 [Ref. 59] Direct ncRNA 10.1093/humrep/deaa184; PMID 32914196	Retrieval prognosis; multivariable prediction model (PROBAST+AI-relevant); timing NR	Frame: other: selected outcome-defined prognostic development cohort; consecutive status NR; centers: 3 centers; recruitment NR; participants: Discovery 6+5; model training: n=30; held-out validation: n=66 with NOA	Nine-lncRNA EV panel transferred to held-out validation; no geographic lock/replication; selection: post hoc/data-derived (discovery plus LASSO)	Sperm retrieval at first micro-TESE; level: patient important procedural/decision outcome; reference/endpoint: testicular sperm presence under the study retrieval pathway	Development plus internal held-out split Independence within report: Participant-independent internal split; not geographically external Performance/precision: Train AUC 0.986 (95% CI 0.956–1.000); validation 0.960 (0.915–1.000); calibration NR	[59]/[54] Sun Yat-sen program; same-program and overlapping investigator clues; participant overlap unclear	Comparator: Same-patient hormone-model comparisons; no full model alone versus plus-panel analysis Concern: Strong internal signal but single program, selected case mix and incomplete incremental-value test Audit note: Treat the validation partition as internal and do not assume independence from [54].
Chen 2021 [Ref. 54] Direct ncRNA 10.1007/s10815-021-02101-8; PMID 33686546	Retrieval prognosis; small prediction-model development/validation; timing NR	Frame: other: selected NOA development cohort with comparator groups; consecutive status NR; centers: 1 clinical center; recruitment: January–December 2019; participants: training, n=20; validation, n=25 at one center	EV piR-61927 model; threshold/model not geographically locked; selection: post hoc/data-derived (sequencing, verification, and LASSO)	Sperm retrieval at micro-TESE; level: patient-important procedural/decision outcome; reference/endpoint: micro-TESE retrieval outcome	Development plus same-center internal validation Independence within report: Participant-independent internal groups; not external Performance/precision: AUC 0.82 (95% CI 0.63–1.00) and 0.83 (0.66–1.00); calibration reported	[59]/[54] Sun Yat-sen program; same-program and author overlap; participant overlap unclear	Comparator: No full contemporary clinical comparator Concern: Very small samples and wide uncertainty despite reported calibration Audit note: The 25-patient validation group is same-center internal validation.

Study / evidence role	Intended use / design timing	Sampling frame / setting	Analyte / selection	Outcome / level	Validation / performance	Cohort independence	Current-care comparison / principal limitation
Ji 2021 [Ref. 61] Direct ncRNA 10.1093/humrep/deab196; PMID 34477868	Retrieval prognosis; biomarker-panel development/apparent performance; retrospective timing	Frame: other: retrospective selected cohort; consecutive status NR; centers: 1; recruitment N; participants: feature discovery, n = 6 testes; the 52-patient cohort was used for model development/evaluation within one program	Three seminal-plasma circRNAs; combined panel selected during development; selection: post hoc/data-derived (including LASSO)	Sperm retrieval at micro-TESE; level: patient-important procedural/decision outcome; reference/endpoint: micro-TESE outcome	Development/apparent performance; no clean independent validation Independence within report: Not independent from model selection/evaluation Performance/precision: Combined AUC 0.958; AUC CI and calibration NR	None established; no cross-report reuse established; within-report evaluation is not an independent holdout	Comparator: No complete clinical-model comparison Concern: The same 52-patient cohort was used for model development/evaluation; no independent validation or precision estimate Audit note: Use “apparent/internal performance,” not external validation.
Zhang 2022 (four-miRNA panel) [Ref. 53] Direct ncRNA 10.21037/atm-21-5100; PMID 35530943	Retrieval prognosis; multivariable miRNA model (PROBAST+AI-relevant); retrospective timing	Frame: case–control /outcome-defined retrospective groups; consecutive status NR; centers: 1 clinical program; recruitment NR; participants: screen, n=18; train, n=56; blinded test, n=40.	Four-miRNA panel evaluated in a blinded internal test set; selection: post hoc/data-derived	Sperm retrieval at micro-TESE; level: patient-important procedural/decision outcome; reference/endpoint: micro-TESE retrieval outcome	Development plus blinded internal test set Independence within report: Participant-independent internal test set; no external geography/program Performance/precision: Test AUC 0.927; AUC CI and calibration NR	None established; no participant overlap established with other S1 reports	Comparator: No full clinical model alone versus plus-panel comparison Concern: No geographic validation; precision and calibration insufficiently reported Audit note: Blinding strengthens the internal test but does not make it external validation.
Zhang 2022 (two EV-tRFs) [Ref. 56] Direct ncRNA 10.1016/j.fertnstert.2021.11.010; PMID 34955241	Retrieval prognosis; small exploratory diagnostic/prediction study; prospective timing	Frame: case–control /selected retrieval groups and fertile controls; consecutive status NR; centers: 1 clinical center; recruitment NR; participants: 12 retrieval-positive, 18 retrieval-negative and 12 fertile controls	Two circulating EV tRFs, reported as exosomal; cut-offs derived in small selected sample; selection: post hoc/data-derived	Sperm retrieval at micro-TESE; level: patient-important procedural/decision outcome; reference/endpoint: micro-TESE retrieval plus fertile-control contrast	Development/apparent performance; no independent validation set Independence within report: Discovery subset reused in the evaluation dataset Performance/precision: AUCs 0.921 and 0.954; AUC CIs/calibration NR	[55]/[52]/[56] Xuzhou-associated program; same center/author as [55]; participant overlap unclear	Comparator: No clinical comparator Concern: Very small case–control sample and mixed retrieval/fertile contrasts Audit note: Do not count the qPCR cohort as wholly independent of the sequencing discovery subset.
Han 2022 [Ref. 55] Direct ncRNA 10.1186/s12958-022-00978-3; PMID 35869479	OA/NOA classification and retrieval prognosis; two-stage biomarker study; timing NR	Frame: case–control /selected diagnostic and retrieval groups; consecutive status NR; centers: 1; recruitment NR; participants: 18 retrieval-positive and 23 retrieval-negative NOA men plus comparator groups	EV tRF-Val-AAC-010; intended-use-specific thresholds require separation; selection: post hoc/data-derived	OA/NOA classification and micro-TESE retrieval; level: mixed—intermediate diagnostic label plus patient-important procedural outcome; reference/endpoint: origin classification and micro-TESE retrieval are distinct outcomes	Development/internal only Independence within report: No external validation; relationship to [56] participants unclear Performance/precision: Retrieval AUC 0.89, sensitivity 72%, specificity 91%; CI/calibration NR	[55]/[52]/[56] Xuzhou-associated program; same center/authors as [56]; participant overlap unclear	Comparator: No full clinical comparator Concern: Risk of transferring the higher OA/NOA AUC (0.96) incorrectly to retrieval prognosis Audit note: Keep the OA/NOA AUC separate from the lower retrieval-prediction AUC.
Lv 2024 [Ref. 62] Direct ncRNA 10.1111/andr.13617; PMID 38421140	Retrieval prognosis; serum circRNA prediction model (PROBAST+AI-relevant); prospective timing	Frame: other: prospective NOA cohort undergoing micro-TESE; consecutive status NR; centers: 1; recruitment: July 2020–March 2022; participants: a 20-sample screening subset drawn from the same 180-patient dataset (84 SR+, 96 SR−)	Six-circRNA serum panel; no independent locked-assay replication; selection: Post hoc/data-derived	Sperm retrieval at micro-TESE; level: patient-important procedural/decision outcome; reference/endpoint: testicular sperm retrieval outcome	Development/apparent performance; no untouched holdout Independence within report: Full-cohort reuse of screening participants Performance/precision: AUC 0.977, sensitivity 91.7%, specificity 86.5%; AUC CI/calibration NR	[62]/[63] Northwest Women’s and Children’s Hospital program; same center and overlapping recruitment period with [63]; participant overlap unclear	Comparator: No nested contemporary clinical comparison Concern: High-dimensional selection and evaluation reused the same dataset; no clean internal or geographic validation Audit note: The 20-patient screen is not an independent cohort from the 180-patient evaluation.
Shi 2023 [Ref. 63] Direct ncRNA 10.3389/fendo.2022.1109807; PMID 36733803	Retrieval-outcome association and internal prediction model; retrospective single-center study; retrospective timing	Frame: other: retrospective iNOA cohort; consecutive status NR; centers: 1; recruitment: January 2017–November 2021; participants: 114 men with idiopathic NOA undergoing micro-TESE; training, 58; same-center validation, 56.	circ_*MGLL* in paraffin-embedded tissue obtained during micro-TESE; nomogram with FSH, luteinizing hormone, testosterone and postoperative pathological type; no locked preoperative assay; selection: post hoc/model-derived	Sperm retrieval at micro-TESE; predictor set includes testicular tissue/pathology acquired during the procedure; level: patient-important procedural outcome, but the model is not purely preoperative; reference/endpoint: sperm retrieval under the study micro-TESE pathway	Development plus same-center internal split/bootstrapping Independence within report: Participant-independent internal split; not external Performance/precision: AUC 0.868 (reported 95% limits 0.776–0.960) training and 0.811 (0.701–0.922) validation; calibration and decision curves reported; source-table upper/lower labels are transposed	[62]/[63] Northwest Women’s and Children’s Hospital program; same center and overlapping recruitment window as [62]; participant overlap unclear	Comparator: Combined model compared with circ_*MGLL* alone, not a prespecified preoperative current-care model with versus without ncRNA Concern: Molecular and histological predictors unavailable before the procedure; retrospective single center; no geographic validation Audit note: Do not describe the nomogram as wholly noninvasive/preoperative because tissue/pathology information is procedural.
Babakhanzadeh 2026 (circ_0070963 axis) [Ref. 64] Direct ncRNA 10.18502/ijrm.v24i3.21113; PMID 42200093	Retrieval prognosis / molecular axes; case–control model development; timing NR (case-control design is reported; timing orientation is not)	Frame: case–control /selected 60 NOA patients and 40 fertile controls; consecutive status NR; centers: 1 recruitment center; recruitment: July–September 2025; participants: each report describes 60 NOA and 40 fertile men with closely matched authorship/cohort structure	A novel hsa_circ_0070963/miR-223-3p/*CCDC96*-*CCDC112* regulatory axis as a non-invasive biomarker for predicting sperm retrieval outcomes in non-obstructive azoospermia: a case–control study; selection: post hoc/data-derived	Sperm retrieval at bilateral micro-TESE; level: patient-important procedural/decision outcome; reference/endpoint: retrieval outcome within NOA alongside fertile-control contrasts	Development/apparent performance; no separate validation cohort Independence within report: Same participant cohort as [65] and [66]; not independent Performance/precision: Reported retrieval AUCs 0.920–0.983; CIs/calibration NR	[64–66] Babakhanzadeh 60-NOA/40-control cohort; confirmed same cohort across all three reports	Comparator: No complete clinical comparator Concern: Possible participant overlap, extreme controls and non-independent reports Audit note: Count [64–66] as three molecular analyses of one participant cohort, not three independent cohorts.

Study / evidence role	Intended use / design timing	Sampling frame / setting	Analyte / selection	Outcome / level	Validation / performance	Cohort independence	Current-care comparison / principal limitation
Babakhanzadeh 2026 (*INPP1*/*GTSF1*) [Ref. 65] Direct ncRNA 10.1038/s41598-026-47513-7; PMID 42162075	Retrieval prognosis / molecular axes; case–control model development; timing NR	Frame: case–control /selected 60 NOA patients and 40 fertile controls; consecutive status NR; centers: 1 recruitment center; recruitment: NR in this report; participants: each report describes 60 NOA and 40 fertile men with closely matched authorship/cohort structure	Uncovering hidden spermatogenesis: *INPP1* and *GTSF1* as non-invasive predictors of sperm retrieval in non-obstructive azoospermia; selection: post hoc/data-derived	Sperm retrieval at bilateral micro-TESE; level: patient-important procedural/decision outcome; reference/endpoint: retrieval outcome within NOA alongside fertile-control contrasts	Development/apparent performance; no separate validation cohort; independence within report: same participant cohort as [64] and [66]—not independent; performance/precision: reported retrieval AUCs of 0.920–0.983; CIs/calibration NR	[64–66] Babakhanzadeh 60-NOA/40-control cohort; confirmed same cohort across all three reports	Comparator: no complete clinical comparator; concern: possible participant overlap, extreme controls and non-independent reports; audit note: recruitment dates remain NR in [65]; timeline audit concern: [65] was received 24 August 2025, before the end of the July–September 2025 window reported for the identical cohort in [64]; record as a date-reporting inconsistency, not as a replacement date.
Babakhanzadeh 2026 (circ_0081481 axis) [Ref. 66] Direct ncRNA 10.1038/s41598-026-49620-x; PMID 42168344	Retrieval prognosis / molecular axes; case–control model development; timing NR	Frame: case–control /selected 60 NOA patients and 40 fertile controls; consecutive status NR; centers: 1 recruitment center; recruitment NR in this report; participants: each report describes 60 NOA and 40 fertile men with closely matched authorship/cohort structure	Hsa-circ_0081481-miR 3960-*FBXO24* regulatory axis in non-obstructive azoospermia identifies potential biomarkers of spermatogenic failure; selection: post hoc/data-derived	Sperm retrieval at bilateral micro-TESE; level: patient-important procedural/decision outcome; reference/endpoint: retrieval outcome within NOA alongside fertile-control contrasts	Development/apparent performance; no separate validation cohort; independence within report: same participant cohort as [64] and [65]—not independent; performance/precision: reported retrieval AUCs of 0.920–0.983; CIs/calibration NR	[64–66] Babakhanzadeh 60-NOA/40-control cohort; confirmed same cohort across all three reports	Comparator: no complete clinical comparator; concern: possible participant overlap, extreme controls and non-independent reports; audit note: recruitment dates remain NR in [66]; timeline audit concern: [66] was received 16 July 2025, before the end of the July–September 2025 window reported for the identical cohort in [64]; record as a date-reporting inconsistency, not as a replacement date.
Xi 2026 [Ref. 92] Clinical-only benchmark 10.1111/andr.70114; PMID 40916621	Clinical retrieval-prediction model; multivariable/ML (PROBAST+AI-relevant); timing NR	Frame: other: multicenter NOA cohort; consecutive status NR; centers: multicenter; exact number NR in accessible primary abstract; recruitment: NR in accessible primary abstract; participants: >2800 men across centers; includes external cohort	Routine-variable model; model specification should be checked at implementation; selection: post hoc/model-derived	Sperm retrieval at micro-TESE; level: patient-important procedural/decision outcome; reference/endpoint: micro-TESE retrieval outcome	Development plus internal and external validation; independence within report: external validation reported; exact geographic/temporal details NR in accessible abstract; performance/precision: internal AUC of 0.8469; external AUC of 0.8301; CIs NR in the reviewed report	Non-molecular comparator evidence unit; no overlap with molecular cohorts established	Comparator: research benchmark using routine variables; not a universally adopted calculator or same-patient ncRNA comparison; concern: different population spectrum/design makes its AUC non-comparable with selected case–control ncRNA estimates; audit note: use only as a non-molecular benchmark; do not merge its validation status with ncRNA evidence.
Jodar 2015 [Ref. 77] Contextual mixed RNA 10.1126/scitranslmed.aab1287; PMID 26157032	Adjacent mixed coding/non-coding sperm-RNA prognostic comparator; couple/pathway outcome study; retrospective timing	Frame: other: selected infertility-treatment/couple pathway cohort; consecutive status NR; centers: 2 collection centers; recruitment NR; participants: 96 idiopathic infertile couples	648 sperm RNA elements, predominantly exonic (585) with 42 non-coding; independently locked validation absent; selection: post hoc/data-derived SRE selection	Live birth after fertility treatment; level: patient-important outcome; reference/endpoint: live birth after timed intercourse/IUI; treatment-pathway-dependent	Development/internal assessment plus small independent-clinic test subset; not a clean, locked external validation; independence within report: partial independent-clinic testing, but model/feature lock and full external validation are not established; performance/precision: 73% versus 27% live birth; precision/calibration NR	Possible CReATe program relationship with [80]; same center/senior investigator clues; participant overlap unclear and likely different-era	Comparator: pathway comparison described, but no biomarker-guided management trial; concern: predominantly exonic signature; contextual rather than primary ncRNA evidence; no independent replication; audit note: contextual mixed-RNA study: 585 exonic, 42 noncoding, 12 intronic, and 9 intergenic SREs.
Li 2026 [Ref. 67] Direct ncRNA 10.1007/s10815-026-03940-z; PMID 42430032	ART outcome association; retrospective cohort Timing: Retrospective	Frame: other: selected retrospective IVF cohort; consecutive status NR; centers: 1; recruitment: 2022–2023; participants: 135 IVF couples	Sperm 28S rRNA fragments; no prespecified clinical decision threshold; selection: mixed—targeted candidates from prior literature; 28S finding selected within study; formal preregistration NR	High-quality day-3 embryo rate (primary molecular association); clinical pregnancy also recorded; level: primarily intermediate embryology outcome; reference/endpoint: embryo quality (intermediate endpoint)	Exploratory targeted association/internal analysis; no external validation; independence within report: single-cohort performance/precision: association reported; decision accuracy/calibration not applicable or NR	None established; no cross-report cohort reuse established	Comparator: conventional semen measures considered, not a complete clinical decision model; concern: retrospective intermediate outcome; couple/cycle confounding and no utility evaluation; audit note: do not elevate embryo-quality association with live-birth prediction.
Al Smadi 2026 [Ref. 83] Direct ncRNA 10.1016/j.ncrna.2025.09.008; PMID 41312059	ART outcome biomarker discovery with follow-up RT-qPCR; timing NR	Frame: other: selected infertility-treatment discovery and validation cohorts; consecutive status NR; centers: 1 infertility center; recruitment NR; participants: 13 discovery donors (39 individually selected sperm libraries) plus 85 bulk-sperm validation men	Selected miRNAs; sequencing and validation compartments differ; selection: post hoc/data-derived	Embryo quality plus β-hCG, clinical pregnancy, and live birth; level: mixed—intermediate embryology and patient-important pregnancy/live-birth outcomes; reference/endpoint: embryo quality, β-hCG and live-birth associations	Development plus participant-independent same-center validation; measurement compartment changes; independence within report: participant-independent but not external; validation changes from individually selected sperm to bulk sperm; performance/precision: combined AUC of 0.75; CI/calibration NR	No participant overlap established; investigator lineage with [48] only; no cohort reuse established	Comparator: female/cycle factors incompletely incorporated; concern: small discovery unit, multiple outcomes and possible measurand shift between selected and bulk sperm; audit note: call the 85-man cohort “same-center participant-independent validation”, not external validation.

Study / evidence role	Intended use / design timing	Sampling frame / setting	Analyte / selection	Outcome / level	Validation / performance	Cohort independence	Current-care comparison / principal limitation
Huang 2026 [Ref. 45] Direct ncRNA 10.1016/j.gendis.2025.101807; PMID 42011481	Semen-category classification; small prospective assay-development study; prospective timing	Frame: other: selected semen-phenotype strata; consecutive status NR; centers: 1; recruitment NR; participants: 25 men (9 normozoospermia, 9 asthenozoospermia and 7 teratozoospermia)	PANDORA-seq tsRNA and rRNA-derived signatures; features selected within the same program; no externally locked assay; selection: post hoc/data-derived	Sperm-quality/semen phenotype classification; level: intermediate/diagnostic label; reference/endpoint: World Health Organization semen-category labels	Development plus random 70:30 internal split; independence within report: internal split only; performance/precision: AUC ≥ 0.83; confidence intervals and calibration not reported	None established; no cross-report cohort reuse established	Comparator: reproduces semen-analysis categories; no clinical incremental-value comparison; concern: very small cohort and held-out subset; no external validation; the endpoint is not infertility, ART outcome or clinical utility; audit note: the 70:30 split is internal and very small.
Hamilton 2022 [Ref. 81] Direct ncRNA 10.1038/s41598-022-24568-w; PMID 36411317	ART intermediate-outcome discovery study; timing NR	Frame: other: selected donor-oocyte cycles and blastocyst-outcome strata; consecutive status NR; centers: 1; recruitment: April 2017–August 2020; participants: 54 normozoospermic men recruited and sequenced in young-donor-oocyte cycles; analyzed groups: high, 18; low, 14; average, 12; the report does not account for the remaining men	Sperm small-RNA sequencing; no locked panel or clinical threshold; selection: post hoc/data-derived differential-expression analysis	Blastocyst development rate; level: intermediate embryology outcome; reference/endpoint: blastocyst rate, an intermediate ART endpoint	Discovery/association only; no validation cohort; independence within report: single cohort; performance/precision: 324 small RNAs differentially expressed between high and low groups (221 and 84 for average versus high and average versus low); no validated classifier, calibration or deployment precision	Possible CReATe program relationship with [76]; same center/senior investigator; participant overlap unclear	Comparator: Donor oocytes reduce female-factor heterogeneity, but no clinical model comparison; concern: high-dimensional discovery, contrasting groups and no independent validation; audit note: fifty-four men were recruited, but the reported high/low/average analyzed groups total 44; disposition of 10 is not clearly reconciled.
Grosso 2021 [Ref. 57] Direct ncRNA 10.1093/molehr/gaab017; PMID 33693947	ART-outcome association; prospective donor-oocyte ICSI study; prospective timing	Frame: other: selected normozoospermic men in donor-oocyte ICSI/repeated-failure strata; consecutive status NR; centers: NR in accessible primary abstract; recruitment NR; participants: normozoospermic men undergoing donor-oocyte ICSI; comparison with repeated failed cycles (sample size not stated in the accessible record)	Selected seminal-plasma tRFs; no locked multivariable model or threshold reported; selection: targeted tRF candidates; formal prespecification/preregistration NR	ART/ICSI success and repeated failed cycles; level: patient-important treatment-pathway outcome (live birth not explicitly established in accessible abstract); reference/endpoint: repeated failed ICSI cycles and ART outcomes	Exploratory association; no validation cohort described; independence within report: single study pathway; external independence NR; performance/precision: no deployable AUC, calibration or independently validated threshold reported	None established; no cross-report cohort reuse established	Comparator: No complete same-patient current-care model comparison; concern: association does not establish prediction, incremental value or clinical utility; audit note: sample size is intentionally NR here; a secondary-review count was not substituted for unavailable primary reporting.
Hekim 2025 [Ref. 58] Direct ncRNA 10.1186/s12610-025-00283-0; PMID 40890583	Oligozoospermia classification and exploratory ART-outcome association; timing NR	Frame: other: comparative infertile cohort stratified by sperm concentration; consecutive status NR; centers: 1; recruitment: September 2023–February 2025 Participants: 48 infertile men (23 oligozoospermic and 25 normozoospermic; infertile comparators, not fertile controls)	Seminal 5′tRF-Glu-CTC RT-qPCR; no locked decision rule; selection: named candidate assessed a priori; formal protocol/preregistration NR	Semen category, fertilization/embryo formation, and pregnancy; ART associations were not significant; level: mixed—intermediate semen/embryology and patient-important pregnancy outcome; reference/endpoint: Semen-category contrast; two-pronuclear oocyte, embryo formation and pregnancy outcomes	Exploratory association; no separate validation cohort; independence within report: single cohort performance/precision: group difference reported ($P = 0.024$); ART associations not statistically significant; no calibration or validated threshold	None established; no cross-report cohort reuse established	Comparator: No clinical model alone versus model plus tRF comparison; concern: small selected groups and no external validation; despite the title, no ART-outcome prediction is demonstrated; audit note: the paper's “randomization” wording refers to group allocation by sperm concentration, not a randomized intervention.

Post-search update: Ref. 72 (PMID 42623004; DOI 10.1007/s10815-026-04013-x) was available only at the abstract level. It reports related plasma ceRNA-network predictors from the same investigative network. Sample size, center, recruitment dates, protocol identifiers, and independent validation were not reported in the accessible abstract. It is therefore treated as a same-program update with unresolved participant independence, not as a new independent cohort.

Table S2. Cohort-overlap and evidence-unit map

The comparisons below distinguish report count from independent participant evidence. A confirmed or likely relationship changes how replication is counted; it does not invalidate the molecular analysis.

Evidence family	Reports compared	Relationship	Overlap evidence	Independence conclusion	Analytic handling
[49–51] Fundació Puigvert/IDIBELL	[49] Barceló 2018; 10.1093/humrep/dey072 vs [50] Plata-Peña 2023; 10.1111/andr.13286	Same-program follow-up; direct participant reuse unresolved	Bassas and Larriba shared; Plata-Peña/López-Rodrigo join later work; Both selected men referred to Andrology Service, Fundació Puigvert; IDIBELL collaboration; No exact cross-paper individual or subgroup fingerprint established in available comparison; [49] NR; [50] January 2016–December 2019; Related seminal EV-miRNA program; [50] explicitly validates miR-31-5p derived from [49]	Not geographically/program independent; participant-level overlap unclear	Treat as the same program/evidence lineage; do not claim independent external validation; do not assert participant duplication without further identifiers Certainty: High for same program; unclear for participant reuse.
[49–51] Fundació Puigvert/IDIBELL	[49] Barceló 2018; 10.1093/humrep/dey072 vs [51] Larriba 2024; 10.1111/andr.13461	Confirmed participant reuse	Larriba and Bassas shared; Same Andrology Service, Fundació Puigvert program; Two OA-N individuals have near-identical unique multi-field profiles: age 44, testes 0/15 mL, FSH 8.4/8.38, TESE 0.5; and age 33, testes 0/20 mL, FSH 6.6, TESE 0.182/0.184; NR in both reports; Same azoospermia program and biospecimen type	Not participant-independent	Count as overlapping reports from one program/cohort lineage, not two independent cohorts Certainty: Confirmed by near-exact individual fingerprints (rounding explains 8.4/8.38 and 0.182/0.184).

Evidence family	Reports compared	Relationship	Overlap evidence	Independence conclusion	Analytic handling
[49–51] Fundació Puigvert/IDIBELL	[50] Plata-Peña 2023; 10.1111/andr.13286 vs [51] Larriba 2024; 10.1111/andr.13461	Confirmed substantial participant reuse	Larriba, Bassas, and López-Rodrigo shared; same andrology service, Fundació Puigvert program; exact subgroup matches: Sp+ n=16, age 42.4±7.28, FSH 23.45±11.02; OA-N n=9, age 39.67±4.69, FSH 4.3±2.5. The no-TESE SA group also matches (n=10; near-identical FSH after rounding). Sp− is n=17 in [50] and n=16 in [51], with closely matching age/FSH, consistent with one exclusion/drop (inference); [50] January 2016–December 2019; [51] NR in article; same referral service and azoospermia subgroup definitions	Not participant-independent	[51] calls its RT-qPCR samples independent relative to its own sRNA-seq discovery samples, but they substantially reuse [50]. Do not treat [51] as external to [50] or infer [49]↔[50] reuse transitively Certainty: Confirmed substantial reuse.
[64–66] Babakhanzadeh 60-NOA/40-control cohort	[64] circ_0070963 axis; 10.18502/ijrm.v24i3.21113 vs [65] *INPP1*/*GTSF1*; 10.1038/s41598-026-47513-7	Same participant cohort	Ten shared authors; [65] adds Esmaeili Dahaj and Hakimi; Same Yazd recruitment program and Shahid Beheshti collaboration; exact: 60 NOA + 40 fertile; SCOS/HYPO/MA n=20 each; retrieval: 29 positive/31 negative; positive subtype counts: 3/16/10; ~3 mL blood; identical LH/FSH/testosterone means across all four groups; [64] July–September 2025; [65] NR in report; same ethics IR.SBMU.MSP.REC.1403.331 and bilateral micro-TESE protocol	Not independent	One cohort, two molecular analyses; never count as two independent cohorts Certainty: Confirmed / effectively certain.
[64–66] Babakhanzadeh 60-NOA/40-control cohort	[64] circ_0070963 axis; 10.18502/ijrm.v24i3.21113 vs [66] circ_0081481 axis; 10.1038/s41598-026-49620-x	Same participant cohort	Ten shared authors; [66] adds Esmaeili Dahaj and Solatzadeh; Same Yazd recruitment program and Shahid Beheshti collaboration; exact: 60 NOA + 40 fertile; SCOS/HYPO/MA n=20 each; retrieval: 29 positive/31 negative; positive subtype counts: 3/16/10; ~3 mL blood; identical LH/FSH/testosterone means across all four groups; [64] July–September 2025; [66] NR in report; same ethics IR.SBMU.MSP.REC.1403.331 and bilateral micro-TESE protocol	Not independent	One cohort, two molecular analyses; never count as two independent cohorts Certainty: Confirmed / effectively certain.
[64–66] Babakhanzadeh 60-NOA/40-control cohort	[65] *INPP1*/*GTSF1*; 10.1038/s41598-026-47513-7 vs [66] circ_0081481 axis; 10.1038/s41598-026-49620-x	Same participant cohort	Ten shared authors plus one report-specific collaborator in each; same Yazd recruitment program and Shahid Beheshti collaboration; exact 60/40 total, 20/20/20 histology, 29/31 retrieval, 3/16/10 positive subtype pattern, blood volume, and protocol; NR in both reports; [64] identifies the common cohort window as July–September 2025; timeline concern: [66] received 16 July 2025 and [65] received 24 August 2025, both before that window ended; same ethics IR.SBMU.MSP.REC.1403.331 and bilateral micro-TESE protocol	Not independent	One cohort, two molecular analyses; never count as two independent cohorts Certainty: Confirmed / effectively certain.
[64–66] Babakhanzadeh program plus 2026 update	[64] circ_0070963 axis; 10.18502/ijrm.v24i3.21113 vs Update PMID 42623004; 10.1007/s10815-026-04013-x	Same program/authors; cohort relationship unresolved	Babakhanzadeh, Khodadadian, Nazari, Mozhdeh, Vahidi, Jafari, Dehghani, Ghafouri-Fard, and Ghasemi shared; highly likely the same research program from author team; participant center NR in update abstract; update abstract reports no n, histology counts, retrieval split, blood volume, or ethics ID; [64] July–September 2025; update NR; NR in update abstract	Unclear; not eligible to count as independent without full-text proof	Abstract-level update only; do not pool/count as a new cohort Certainty: High for same program; unclear for participant reuse.
[64–66] Babakhanzadeh program plus 2026 update	[65] *INPP1*/*GTSF1*; 10.1038/s41598-026-47513-7 vs Update PMID 42623004; 10.1007/s10815-026-04013-x	Same program/authors; cohort relationship unresolved	Nine core authors shared; update omits Esmaeili Dahaj/Hakimi and adds no cohort fingerprint in abstract; highly likely the same research program from author team; participant center NR in update abstract; update abstract lacks sample counts and protocol identifiers; [65] NR; update NR; NR in update abstract	Unclear; not eligible to count as independent without full-text proof	Abstract-level update only; do not pool/count as a new cohort Certainty: High for same program; unclear for participant reuse.
[64–66] Babakhanzadeh program plus 2026 update	[66] circ_0081481 axis; 10.1038/s41598-026-49620-x vs Update PMID 42623004; 10.1007/s10815-026-04013-x	Same program/authors; cohort relationship unresolved	Nine core authors shared; update omits Esmaeili Dahaj/Solatzadeh and gives no cohort fingerprint in abstract; highly likely the same research program from author team; participant center NR in update abstract; update abstract lacks sample counts and protocol identifiers; [66] NR; update NR; NR in update abstract	Unclear; not eligible to count as independent without full-text proof	Abstract-level update only; do not pool/count as a new cohort Certainty: High for same program; unclear for participant reuse.

Interpretive summary

The 23 report-level entries do not represent 23 independent cohorts. Refs. 64–66 use the same 60-NOA/40-control cohort. Refs. 50 and 51 show substantial participant reuse, and Refs. 49 and 51 show partial reuse. Other same-program relationships remain unresolved because participant identifiers or complete recruitment windows were unavailable. Internal splits, resampling, and same-center follow-up were not classified as external geographic validation.

Abbreviations: ART, assisted reproductive technology; AUC, area under the receiver operating characteristic curve; CI, confidence interval; EV, extracellular vesicle; FSH, follicle-stimulating hormone; ICSI, intracytoplasmic sperm injection; IUI, intrauterine insemination; micro-TESE, microdissection testicular sperm extraction; ncRNA, non-coding RNA; NOA, non-obstructive azoospermia; NR, not reported; OA, obstructive azoospermia; SR+/SR−, successful/failed sperm retrieval.
